# Enhancing audit quality and reducing costs: the impact of AI in banking and financial services

**DOI:** 10.3389/frai.2025.1718854

**Published:** 2026-01-23

**Authors:** Amar Johri, Anu Sayal, Kim Mee Chong, Maysoon Khoja, Chaithra N, Janhvi Jha, Neha Tyagi

**Affiliations:** 1 College of Administrative and Financial Sciences, Saudi Electronic University, Riyadh, Saudi Arabia; 2 Faculty of Business and Law, School of Accounting and Finance, Taylor’s University, Subang Jaya, Malaysia; 3 Department of CSE (AI & ML), JAIN (Deemed to be University), Bangalore, Karnataka, India; 4 Department of Computer Science and Engineering, Amity University, Noida, Karnataka, India

**Keywords:** artificial intelligence, auditing, banking and financial services, lead propensity analysis, business volume forecasting

## Abstract

**Introduction:**

Auditing methods have been significantly influenced by the combination of automation with artificial intelligence (AI) and have, in part, changed the roles of auditors and the quality of audits. Sampling-based traditional auditing has several challenges with identifying anomalies or new risks in financial information environments that are becoming increasingly complex and more data-rich.

**Methods:**

In this study, AI-based techniques (machine learning and natural language processing) will be applied to a number of the steps involved in auditing financial information. The predictive model will be applied to lead propensity analysis and business volume forecasting, allowing the examination of both structured and unstructured data on financial statements and protecting the privacy of client data.

**Results:**

A predictive model utilizing artificial intelligence (AI) was able to identify leads at an 87% rate of accuracy; forecasted business volume errors were less than 5%; and it explained nearly 94% of the variance between the AI model’s predictions and the actual loan disbursement amounts. Using AI for full dataset analysis instead of sample-based methods improved auditors’ ability to detect anomalies and allocate resources efficiently.

**Discussion:**

Overall, the research demonstrates that AI provides auditors the capability to evaluate all data for a company, automate routine tasks, and identify specific areas (high-risk/high-value) that may require further review compared to other auditing methods. The new methodology also allows for early identification of potential risks and improves the overall efficiency of audits without compromising the protection of the companies’ data.

## 1 Introduction

The auditing and advisory industries have historically relied on manual processes and periodic reviews to assess financial performance, compliance, and risk. However, with data volumes exploding and business environments growing more complex, auditors are under pressure to deliver insights that are more timely, precise, and strategically useful. A global survey by KPMG (2024) found that 72% of companies are currently selectively using artificial intelligence (AI) in their financial reporting processes. It is expected that adoption may reach 99% within 3 years. Likewise, Gartner (2024) stated that 41% of internal audit teams are already using or plan to use generative AI within the year. This points toward a rapid integration of advanced technologies into audit functions. Traditional audit methods like sampling, document checks, and face to face interviews are no longer enough to detect subtle anomalies and emerging risks (Becker and Olaoye, 2024).

The financial sector is undergoing a similar transition that is reshaping accounting and auditing worldwide through the adoption of artificial intelligence (AI) and data analytics (DA) (Kokina and Davenport, 2017). The world is currently experiencing a rapid transformation as a result of the technological revolution. Auditors can leverage artificial intelligence as a management tool. Consequently, they are able to optimize their productivity and leverage technology to their advantage. Artificial intelligence provides a practical solution in the context of auditing by providing auditors with the requisite data in real-time, enabling them to examine the data more quickly and immediately address any identified risks. This enables them to remain ahead of the curve.

Artificial intelligence simplifies auditing by automating repetitive tasks that auditors would otherwise have to perform manually (Rapoport, 2016; Agnew, 2016). Data analytics tools reveal patterns and trends that can apprise risk assessments, identify outliers, and highlight areas that require further investigation by analyzing vast quantities of data (Ajayi and Akinrinola, 2023). In addition, it functions as a preventative measure to detect and anticipate anomalies before they develop into actual issues (Struthers-Kennedy and Nesgood, 2020). De Angelo suggests that the quality of an audit is determined by the auditor’s capacity to identify and disclose discrepancies in financial statements. When audit quality is high, managers and investors will have improved control and superior financial information. Numerous prior investigations have demonstrated that there is substantial demand for high-quality audits to mitigate information asymmetry and enhance earnings management (Hashem et al., 2023).

The research delineates three principal tensions and shortcomings, especially within the context of developing countries, notwithstanding the increasing focus on AI-driven auditing. Primarily, most research has focused exclusively on developed markets (Kokina and Davenport, 2017; Cooper et al., 2019). This reflects a misunderstanding of how artificial intelligence operates in developing countries, which feature unique policies, differing levels of data digitalization, and diverse legacy technological infrastructures. Odoh et al. (2018) from Nigeria and Lokanan et al. (2019) from Vietnam are notable exceptions; however, their focus is exclusively on adoption determinants rather than on the actual effects on quality or cost. This regional bias diminishes the practicality of AI in markets with limited audit resources.

The cost–benefit analysis of artificial intelligence has revealed inconsistent results within the existing body of research. Cooper et al. (2019) and Huang and Vasarhelyi (2019) highlight significant implementation challenges and delayed return on investment in RPA adoption, whereas Omoteso (2012) emphasizes efficiency gains and cost savings. The primary reason this issue remains unresolved is the lack of evidence supporting concurrent optimization, which is essential for resource-constrained BFSI enterprises in emerging nations. Research generally investigates cost implications and quality improvements independently. Zemánková (2019) advises caution regarding personnel transfer, whereas Munoko et al. (2020) emphasize ethical considerations; nevertheless, there remains a deficiency of quantitative data evaluating these trade-offs in connection with improvements in audit quality.

Ultimately, the progress of cumulative knowledge is impeded by methodological inconsistencies. The utilization of conflicting validation techniques (single holdout versus k-fold cross-validation), diverse dataset sizes (ranging from hundreds to millions of records), and varying performance metrics (accuracy, recall, and F1-score) in machine learning research compromises the validity of cross-study comparisons. Furthermore, existing research overlooks the importance of integrating micro-level transaction analytics with macro-level business forecasting, instead focusing on isolated applications such as fraud detection, contract analysis, or forecasting, rather than providing a comprehensive framework that encompasses all three.

This study intends to demonstrate the potential of AI to enhance decision-making, risk management, and adaptability in today’s complex financial environments. The findings are expected to contribute theoretically by extending the literature on audit quality and digital transformation, particularly by illustrating how AI-driven tools reshape audit methodologies and auditor roles within the Banking, Financial Services, and Insurance (BFSI) sector. On the other hand, the study offers practical contributions by showing how organizations can leverage AI to reduce audit costs, improve resource allocation, and foster proactive anomaly detection. More broadly, the research underscores how auditing is evolving beyond its traditional compliance function into a strategic role that promotes transparency, accountability, and credibility in financial reporting. Despite the growing interest in the field, there are considerable gaps in understanding on AI-driven auditing. The methods for AI application in poor nations are ambiguous, as much research has concentrated on established markets like the United States and Europe. These economies include varied legislative frameworks, demonstrate inconsistent data quality, and have differing technology infrastructures. Moreover, it is troubling that contemporary research focuses either on quality improvements or cost reductions in isolation, neglecting to consider both aspects together. It also focuses solely on certain applications, such as fraud detection or forecasting, rather than the wider environment. Notwithstanding its distinctive attributes, the Indian BFSI industry has garnered very less academic attention. Cultural elements, like cash preferences and joint family financial structures, profoundly impact banking operations, and RBI laws diverge from Western Basel III requirements. The CIBIL credit evaluation system operates differently than the FICO model. Moreover, although the theoretical advantages of AI in auditing have been extensively analyzed, there is a lack of practical data with particular performance measures. This is especially relevant for professionals seeking advice on AI deployment.

This study experimentally illustrates how AI may improve audit quality and concurrently save costs in India’s BFSI industry by addressing these gaps. We provide precise measures (including recall rates, mistake percentages, and explained variation) that facilitate evidence-based decision-making by measuring machine learning efficacy in detecting high-risk leads and forecasting business volumes. Our comprehensive technique correlates individual consumer risks with organizational performance, correlating micro-level lead analytics to macro-level business projections. This differentiates us from existing audit approaches. Our research covers a wide array of subjects: firstly, we validate the impact of extensive dataset analysis on audit risk and theoretically enhance audit quality frameworks to include AI-augmented assurance models; secondly, we empirically establish performance benchmarks for developing-market contexts, attaining an 87% recall rate and a 5% forecasting error; thirdly, we methodically demonstrate privacy-preserving synthetic data techniques for research in confidential sectors; and finally, we provide practical implementation roadmaps for audit firms, banks, and regulatory authorities. We provide tangible, quantifiable data within a specific context, allowing practitioners in poor nations to make educated decisions about AI investments, unlike previous research that just addresses AI’s potential.

## 2 Literature review

### 2.1 Traditional auditing approaches

#### 2.1.1 Introduction and historical background

To ascertain the correctness and adherence to applicable rules and regulations of financial statements, auditors routinely employ traditional auditing techniques, which consist of a set of norms and standards. Over the years, many auditing methodologies have evolved into a formal framework designed to improve the integrity and transparency of governmental documents. They originated in ancient societies where financial transactions were subject to accountability (Smith, 2024). Traditional auditing is characterized by a focus on internal controls, professional skepticism, independence, and ethical conduct such as objectivity, confidentiality, integrity and accountability. By utilizing instruments such as formal processes and compliance audits, auditors must preserve objectivity when conducting an examination of a company’s financial transactions. The audit’s credibility and reliability are enhanced by the systematic examination of financial records, extensive fieldwork, and reliance on historical data that are intrinsic in traditional methodologies (From Ancient Roots to Modern Practices: The Fascinating Timeline of Internal Audit History, 2023; Nasr, 2023).

The foundation of auditing standards may be traced back to the historical value placed on fiscal responsibility. Audits were performed by the governments of Ancient Egypt, Greece, and China to ensure that their financial records were accurate and transparent. Around 3,000 BC, the first Mesopotamian record-keepers began using clay tablets as a basic auditing tool to confirm transactions. The ancient Egyptians established a public accountability system for financial management by designating official scribes to document and authenticate all tax and public works documents (Teck-Heang and Ali, 2008). Due to the complexity of transactions and the growth of enterprises, the requirement for systematic methodologies grew throughout the 18th century Industrial Revolution, which led to the creation of auditing. The formation of accounting associations and institutional norms in the early 20th century helped to standardize auditing as a profession. This, in turn, improved the dependability and uniformity of financial statement reporting (Ajao et al., 2016).

#### 2.1.2 Approaches

System based approach—It is clear that the former prioritizes the testing and evaluation of internal controls to ensure precise financial reporting when comparing system-based and substantive-based auditing methodologies. Using management information, auditors evaluate the client’s internal control system after acquiring a thorough comprehension of the established controls. Auditors will proceed to evaluate and validate the internal controls once they have determined their robustness (Karapetrovic and Willborn, 2001). Although substantive testing continues to be conducted, it is less dependent on transactions than the substantive technique. This allows auditors to more effectively identify and mitigate risks that could lead to inaccuracies in financial reporting (Admin, 2023).

Risk based approach—Risk-based auditing seeks to lower audit risks by focusing on high-risk areas and possible major misstatements, therefore improving audit efficiency. Learning about the client’s business, industry, and internal controls will help an auditor first see possible problems. First identifying areas with a high risk of failure, then developing audit programs especially meant to address those areas, and finally, lowering the amount of effort put in areas with a lower risk of failure helps to allocate resources effectively. Finding any threats that can cause variations in financial accounts is the main objective. This will underline high-risk locations and help auditors to reach audit targets by streamlining their job (Griffiths, 2016; Coetzee, 2010).

Compliance Audits—Compliance auditing is absolutely essential to ensure that a company can produce a verifiable and legitimate audit report that fairly reflects the present situation of affairs in case of litigation about audit policies and regulations. All the stakeholders will thus have a thorough awareness of the present state of the company (Losiewicz-Dniestrzanska, 2015). A compliance audit might look at rules generated from internal policies, plans, and processes or ones imposed by outside laws and regulations. Compliance audits help companies to ensure that rules are followed and help to reduce non-compliance risks (Oladutire and Oladeji, 2020).

Although traditional auditing procedures offer advantages such as meticulous scrutiny and comprehensive coverage, they also have their limitations. Historical data and standardized procedures are frequently the primary focus of conventional auditing processes, which may not offer a comprehensive examination of all unique hazards associated with a firm or sector. Additionally, the emphasis on compliance and comprehensive coverage may result in a decrease in efficiency and an increase in audit expenditures (Debreceny et al., 2005; Al-Khasawneh, 2022).

#### 2.1.3 Audit process

The planning phase entails the establishment of the audit’s direction and the verification of its alignment with the organization’s strategic objectives. Through a risk assessment, stakeholders work together to establish objectives and prioritize the most critical risks. It is imperative to develop a comprehensive audit plan that outlines the approach, timeline, and resource distribution, as it serves as a blueprint for the audit (Elliott et al., 2007). By mitigating distractions, optimizing resource utilization, and increasing the probability of identifying significant issues, comprehensive planning that actively engages stakeholders enhances execution efficiency (AlMomani et al., 2024). This process can be difficult and fraught with obstacles, such as inadequate planning as a result of insufficient information or involvement, which may result in the neglect of hazards. Additionally, the capacity to adjust to new information or organizational changes during the audit may be impeded by inflexible planning (Selisteanu et al., 2015).

The audit involves investigating, writing reports, and client conferring. During fieldwork, auditors follow their plan by gathering and examining data to assess internal control effectiveness and degree of regulatory compliance of a business. Among the several approaches used for this aim are interviews and document assessments. This stage helps one to identify injustice and inefficiency. Maintaining stakeholder responsibility requires honest, objective presentation of the audit’s findings, analysis, and recommendations. Evaluating how effectively those measures worked comes next once the results of the audit have been addressed. This guarantees that the management is maintaining the current improvements and has given the suggestions top attention. This phase promotes a development attitude in the face of possible obstacles such managerial resistance or delayed execution (Elliott et al., 2007).

Rising fraud rates are forcing more stringent auditing rules to increase investor confidence by means of tougher regulations (Hazar, 2021) annual audits in which the present corporate environment does not provide enough sample from auditees This method implies a solution based on continuous auditing. Continuous auditing (Chan and Vasarhelyi, 2018) is the phrase for methods of auditing including constant assessment. The key idea is that, either concurrently or within a very restricted time, a report is generated following an audit of a transaction. Real-time production of the audit report follows recognition of inconsistencies. The first implementation of the continuous audit system calls for time and a high degree of skill. Once again, the same modules might be used to at last lower the expenses of continuous audits (Mokhitli and Kyobe, 2019). Vasarhelyi et al. (2012) detail an ongoing monitoring program housed within a large South American bank. Internal audit tracked over more than 1,400 sites 18 separate key performance indicators (KPIs). After the daily variance data were gathered, the regional managers of the branches were only selectively emailed. These key performance indicators (KPIs) aimed to control overrides, including credit exceeding allowed limits or reversal of particular transaction kinds. Audit activities are carried out almost in real-time. This helps the company to do regular operations’ risk analyses and control of its activities. This benefits annual and interim reports alike (Lee et al., 2014). Internal auditors who do ongoing auditing carefully record business processes. This lets outside auditors have a thorough awareness of the risks and controls of the company. The cooperation of internal and outside auditors helps to raise the caliber of audits by means of skill and knowledge exchange (Weins et al., 2017).

Auditing based on computers has supplanted paper-based auditing. The development of technology has spurred the application of Computer-Assisted Audit Techniques (CAATs), hence improving audit process efficiency and effectiveness. Two main categories under which CAATs fall are audit software and test data. To reproduce client operations including inventory counts (Jaber and Abu Wadi, 2018) and to verify numerical correctness of computations, audit software is used. By allowing auditors to automate tedious activities and concentrate on complex analysis, these tools help to raise the general accuracy and quality of the audit (Ciprian-Costel, 2014). Comprising hardware, software, data organization, and processing techniques, electronic data management (EDM) is a system based on telecommunications and computer technology. EDM has poor traceability among other shortcomings even if it improves data processing consistency over hand-made solutions. By means of several programs catered for diverse data processing activities, Generalized Audit Software (GAS) promotes independent audit testing. GAS, developed by a public accounting firm for multi-year client audits, has great advantages in the auditing process even if it requires large development and maintenance costs (Asniarti and Muda, 2019).

### 2.2 Emergence of AI in auditing

Financial the shift from computer-assisted audit tools (CAATs) to artificial intelligence (AI) is transforming financial data analysis and understanding by auditors. While managing big datasets using manual methods and subsequently Computer-Assisted Audit Techniques (CAATs), auditors encountered issues including time inefficiency and human error. Constant technological progress has resulted in the incorporation of digital technology and data analytics by the auditing field. These tools enhanced financial abnormality and inefficiencies identification ability as well as audit accuracy. The move to continuous auditing—a method using modern technology to examine financial data constantly—showcases the changing dynamics of the vocation. The shift from CAATs to artificial intelligence in auditing is becoming clearer as artificial intelligence grows.

The potential of AI in auditing mostly concentrate on automating monotonous activities. These are systematic, replicable procedures conducted during the audit. AI is anticipated to significantly impact audit duties that are currently performed manually and have received some technological assistance. The influence of artificial intelligence in audits is particularly significant in data collection, encompassing data extraction, comparison, and validation (Zalcewicz, 2023). AI-enabled technology facilitates the identification, extraction, and presentation of pertinent information from documents for human auditors, hence allowing more focus on aspects necessitating advanced judgment (Srinivasan, 2016). AI facilitates the complete automation of labor-intensive operations, such as payment transaction testing, and encompasses the collection of relevant supporting data for more comprehensive analysis. Deloitte, in collaboration with Kira Systems, analyzed material such as contracts, leases, employment agreements, invoices, and other legal data to extract pertinent phrases. Through human interaction, the system acquires proficiency and enhances its capacity over time to collect significant and pertinent data (Whitehouse, 2015).

Using survey data, Odoh et al. (2018) investigated the influence of artificial intelligence on accounting functions. The results suggested that the implementation of AI technology by accounting firms in South East Nigeria resulted in an improvement in their performance. Kokina and Davenport (2017) distinguish four distinct categories of AI applications and an additional four classifications for the various levels of AI that have been developed to date. Numerical analysis, word and image processing, and the execution of both digital and physical tasks are among the applications. Self-aware intelligence, contextual awareness and learning, human aid, and robotic process automation are the four levels of intelligence. At present, there are no AI applications that are capable of self-aware intelligence. However, there is a possibility that significant progress could be made in accounting and auditing with the other three levels of intelligence.

Auditing systems are developing quickly as more companies commit funds to artificial intelligence capabilities. Artificial intelligence (AI) uses historical data from related problems to enable population analysis and enhances decision-making by machine learning. The shift to artificial intelligence might help auditors become strategic partners, therefore improving their value in a market growingly linked by technology. With 58% of financial reporting executives admitting Generative AI (GenAI) as their main technological goal for the next year, Generative AI (GenAI) is becoming more and more important to them (Zulkarnain et al., 2024).

## 3 Key AI technologies applied to auditing

Improvements in artificial intelligence (AI) use in the auditing process might transform the way audits are carried out in several areas. These technologies increase operational accuracy and efficiency, therefore enabling real-time monitoring to ensure regulatory compliance and to enhance auditors’ capacity for substantive reviews. The increasing use of artificial intelligence in audits reveals the general digital transformation trend in the financial services sector. By addressing difficult tasks such risk management and fraud detection, this technology helps to simplify conventional auditing procedures.

### 3.1 Machine learning

Machine learning (ML) was first presented in the 1950s when scientists started experimenting with methods allowing computers to learn and provide predictions based on data (Li, 1959). Machine learning (ML) started to gather pace in several fields, including finance and accounting, as computer capability and data storage capacity grew. Data auditors might use machine learning techniques to more effectively examine massive amounts of data than via hand approaches (Dickey et al., 2019).

Applied for tasks spanning from regression and classification, supervised learning is a basic machine learning technique applied in auditing to educate models on labeled datasets. By use of regression analysis, this enables auditors forecast utilizing historical data by means of anomalies and correlations between financial accounts (Hajek and Henriques, 2017). On the other hand, auditors might apply unsupervised learning—looking at unlabeled data for hidden patterns—to cluster like transactions and spot anomalies free from reliance on established categories. Among other techniques, hierarchical aggregative clustering helps to clarify dangers. Furthermore, more and more auditors are applying reinforcement learning as it enables agents to maximize rewards by means of best interactions with the environment. As I am currently developing it, it might enhance auditing methods by allowing continuous learning and change (Hoogduin, 2019).

Auditing firms are evaluating the potential uses of machine learning within their operations. Deloitte utilizes a machine learning technology called Argus, which is proficient in understanding sales agreements, leases, and contracts, including derivatives. Argus’s technologies enable the identification of trends, deviations, and important contractual conditions (Benkepes, 2016). Analyzing journal entries allows Halo to identify anomalous trends, including submissions from unknown sources, entries that are marginally below the acceptable threshold, or postings containing dubious keywords. Halo improves the efficiency and accuracy of auditing by allowing auditors to review all journal entries of a corporation for a given year and subsequently focus on the most questionable entries (Kokina and Davenport, 2017). Cortex is a cloud-based analytics solution initially developed for audit and tax clients. The application of this approach is increasingly broadening to encompass risk management, consultancy, and financial advisory services. Cortex provides centralized data storage, advanced analytics, models, algorithms, patterns, and data management tools (Artificial Intelligence and Analytics, n.d.). In accordance with auditing standards, executive fraud interviews can be conducted using machine learning technologies, including speech recognition and image analysis (Sun and Vasarhelyi, 2017). Sifa et al. (2019) introduce the Automated List Inspection (ALI) method for the automatic inspection of financial statements, which integrates domain expertise, natural language processing, and machine learning. Ali is a recommendation system that integrates legal requirements with financial statement remarks by taking context into account. This work delineates the architecture of the recommendation system, as well as language modeling, text mining, binary classification models, and supervised and unsupervised approaches. The writers offer recommendations for tool enhancements by assessing algorithms according to both mathematical and subjective criteria. Lokanan et al. (2019) employed machine learning techniques, namely a dynamic anomaly detection algorithm, to evaluate the reliability of quarterly financial reports from all publicly listed Vietnamese companies in order to identify abnormalities in financial statements. The results indicated that the algorithm could evaluate quarterly financial reports in accordance with creditworthiness. According to the implemented model, more than one-fourth of Vietnamese publicly listed firms had dubious financial statements; the majority are reliable. Ding et al. (2019) employed K-medians clustering, a machine learning-based peer selection technique, to analyze significant financial parameters of organizations relevant to specific research objectives. The study employed a dataset comprising 598 bankruptcies and 48,536 non-bankrupt firm-year records, utilizing K-medians clustering with material misstatement detection and bankruptcy prediction algorithms. The study demonstrated that including data on K-medians clustering-based peer businesses using a machine learning approach enhanced the models.

Through comprehensive analysis of whole datasets employing machine learning algorithms (Lam et al., 2024), auditors can eliminate the risk of bias or oversight associated with manual auditing. By employing machine learning models to detect and report abnormalities in real-time, auditors may promptly identify and rectify instances of fraud or errors. Identifying financial data irregularities, questionable transactions, or irregular expenditure patterns is essential (Minkkinen et al., 2022). Organizations may use a proactive fraud detection system to preserve their brand and mitigate financial losses. The scalability and adaptability of machine learning enable audit procedures to align with the requirements of firms in dynamic corporate environments.

Legal and regulatory restrictions often hinder auditors from accessing the large data warehouses of companies such as Google or Facebook. Ethical and client confidentiality concerns might limit auditors’ access to data, therefore restricting the quantity and quality of information available to build their training datasets (Issa et al., 2016).

Machine learning technologies have enhanced the capacity of auditors to assess internal systematic links and external environmental aspects, but they still need a thorough awareness of data intake, processing, and output from many sources. Machine learning technologies may greatly improve auditors’ intuition; yet, to take use of these insights, auditors need to change their perspective. As machine learning (ML) develops, the auditing industry is changing to equip auditors for a data-centric future by generating new job possibilities and improving already existing jobs.

### 3.2 Natural language processing (NLP)

To enable human-like language processing for some applications or activities, NLP—a spectrum of computer techniques for evaluating and presenting texts at various degrees of linguistic analysis—is used. Natural language processing (NLP) is fundamental in auditing as it enables data extraction and analysis from unstructured sources such as contracts and financial statements. Natural language processing helps auditors manage vast textual material, simplifies compliance verification, and enhances document review practices. Auditors should apply sentiment analysis methods to evaluate market opinions and create smart recommendations.

The primary application of natural language processing in audits is the examination of contracts and related documents. Financial auditors conduct a meticulous examination of relevant documents, such as invoices and contracts, to guarantee the accuracy of financial records. AI tools that employ natural language processing (NLP) effectively derive valuable data from these texts and identify any defects or anomalies (Fisher et al., 2016). This reduces the time required for manual document analysis and improves the audit’s accuracy. By employing natural language processing (NLP), auditors can concentrate on critical issues by examining extensive contracts for clauses that may impact financial reporting (How Does AI improve Financial Audits?—Blog, n.d.). Qiu et al. (2014) conducted a TF-IDF analysis, which was further augmented by the LibSVM regression program (SVR) and a linear SVM multi-class classification method, to identify a significant correlation (*p* < 0.001) between subsequent business performance and mandatory annual report disclosures. Predictive analytics in financial forecasting also depend on NLP to be very important. Sentiment analysis techniques allow companies to evaluate the emotional tone of news items, customer evaluations, and social media posts, therefore gaining important insights into consumer satisfaction and market mood (Mitta, 2023). Banks and other financial institutions can utilize this information to improve their assessment of suitable risk management measures, market volatility prediction, and investment possibilities creation (Sousa et al., 2019). Making appropriate investment selections depends on accurate assessment of market trends. Natural language processing (NLP) by use of text data pattern analysis can produce these judgments (Cogent | Blog | NLP and NLG in Finance: Risk Management, Fraud Detection, and Customer Insights, n.d.; Gao et al., 2021). Hagenau et al. (2013) achieved an accuracy of 71.8% in predicting stock-price fluctuations that were signaled by German financial news by utilizing an SVM classifier and bi-normal separation-based feature selection. The “significant negative relationship” between market volatility and news releases from 2000 to 2010 was validated by Smales (2014) using Ravenpack’s Multi-Classifier for Equities mood indicator to measure the implied volatility index.

Purda and Skillicorn (2015) achieved an 89% accuracy rate in the accurate classification of 240 Canadian AAERs as either genuine or fraudulent through natural language processing. In order to differentiate between various interpretations of the same term, the authors employed the Q-Tagger tool, word frequency counts, and part-of-speech (POS) annotation to generate a “rank-ordered list of terms.” They subsequently implemented a Random Forests methodology. The financial misrepresentation F-score from Dechow et al. (2011) was integrated with the analyses provided by an SVM system to verify the veracity of the reports.

### 3.3 Robotic process automation (RPA)

Robotic process automation (RPA) automates repetitious business operations by simulating human interactions with multiple applications or analytics through an interface and making decisions based on fundamental principles (Kaya et al., 2019; Madakam et al., 2019). The banking and financial services industry has been substantially transformed by robotic process automation (RPA), which has improved operational efficiency, accuracy, and compliance in audits. The implementation of RPA in auditing has been delayed due to concerns regarding risk and legislation, despite the fact that tasks such as internal control testing, reconciliations, and detail testing are well-suited for it (Cooper et al., 2019; Moffitt et al., 2018). Adoption of RPA in auditing is low particularly due to the prevalence of outdated legacy systems within the banking sector RPA is being implemented by accounting firms in order to enhance efficiency, minimize expenditures, and establish a competitive edge. Huang and Vasarhelyi (2019) contend that internal audit departments can capitalize on their expertise in RPA to implement automation-enabled controls throughout the organization. In order to optimize their audit procedures, they may implement RPA. Firms may employ RPA to establish a continuous monitoring and auditing program that emphasizes this methodology as agile auditing gains momentum.

Through automation of specific tasks, banks may create significant savings. RPA helps companies to handle more transactions with less staff, therefore lowering labor costs and reallocating personnel to more complex duties (Lamberton et al., 2017). Dilmegani (2024) claims that for financial companies, RPA deployment might save operating expenses by 20–25%. RPA lowers the time spent on manual tasks and boosts efficacy, which produces these results.

### 3.4 Neural networks

Deep learning, a subset of machine learning, allows computers to generate conceptual models of their surroundings and learn from their mistakes. Deep learning is a distinctive approach to evaluating financial services and companies. This method utilizes sophisticated machine learning methodologies to improve the quality, efficacy, and risk management of audits (Sun and Vasarhelyi, 2017). Neural networks are a type of machine learning that allows computers to acquire new tasks by referencing previously taught examples.

Autoencoder neural networks, an unsupervised deep learning technique, have a variety of applications, such as anomaly detection, natural language processing, and image classification. The encoder and decoder are the two components of the traditional autoencoder network, and they both rely on backpropagation. The fundamental concept is encapsulated by the simultaneous training of the encoder and decoder to reduce the discrepancy between the original data and its reconstruction. Kwon et al. (2019) conducted a thorough investigation of deep learning-based network anomaly detection techniques, with a particular emphasis on autoencoders as a method that is particularly promising in comparison to other intrusion detection approaches. In order to identify fraudulent journal entries in extensive accounting data extracted from ERP systems, Schreyer et al. (2017) implemented autoencoder neural networks. After comparing the results of an autoencoder with those of other unsupervised anomaly detection methods, they have determined that it can function as an adaptive anomaly evaluation tool for journal entries. The methods include Principal Component Analysis, One-Class Support Vector Machine, Local Outlier Factor, and Density-Based Spatial Clustering of Applications with Noise (DBSCAN). Nevertheless, the quantitative assessment was contingent upon the inclusion of a small number of intentionally anomalous journal entries in the databases. Models such as Fuzzy Neural Networks outperform conventional statistical methods in the context of planning and executing auditing assignments. This allows auditors to detect financial statements that may contain substantial errors. The enhancement of decision-making across a variety of scenarios is achieved through the application of fuzzy mathematics for representing training samples, the transformation of binary labels into multi-category labels based on membership values, and multi-category decision-making using random forest trees (Algehyne et al., 2022). Zhao et al. 2023 introduce an enterprise financial accounts early warning model that integrates fuzzy sets and random forest trees. The model employs the training prediction sample for evaluation, selection, and initial dataset generation. The cost is applicable regardless of the presence of the ST designation, and the data labels provide supplementary context. Fuzzy mathematical techniques are employed to scramble the training sample data subsequently to the conversion of the binary classification label into a multiclass label. Subsequently, the random forest model is implemented to train the data. Alameer et al. (2019) implemented a particle swarm optimization methodology that was based on soft computing methodologies and incorporated artificial neural networks in order to predict economic volatility. Utilizing a genetic algorithm and an ANFIS, the proposed model is developed. The parameters of the ANFIS model are estimated using genetic algorithms.

Integrating neural networks with knowledge graph technology revolutionizes audits, especially in complex scenarios such as power engineering projects. This configuration enhances auditing efficiency and risk identification by integrating real-time data with predictive analytics (Zhong et al., 2024). Fu et al. (2019) developed a strategy for relation triple extraction via Graph Convolutional Networks (GCNs). The potential to accelerate the processing and retrieval of information from extensive knowledge graphs while reducing the risk of data loss is enabled by the decentralized processing architecture of cloud-edge computing, which enables data to be stored on local devices. In addition, the potential to facilitate real-time data analysis is achieved by substantially reducing latency periods by processing data closer to the source at the network’s edge (Wang and Wan, 2021; Mitropoulou et al., 2024). Edge computing reduces transmission latency and bandwidth requirements by locally processing data, which enhances and modifies auditing. However, the maintenance of data quality remains a significant challenge (Yang et al., 2023).

For its application in auditing, neural networks’ great prediction accuracy above more traditional statistical approaches is a clear benefit. In auditing, neural networks provide advantages in terms of their scalability, adaptation to many data sources, analysis and understanding of complex data patterns, and efficiency.

### 3.5 AI applications in BFSI audit contexts

AI technologies in auditing provide several unique duties depending on the particular area of risk being analyzed. Our first propensity research had an 87% recall rate, demonstrating that machine learning models can proficiently evaluate creditworthiness indicators to pinpoint high-risk portfolios requiring more scrutiny during credit risk audits. Artificial intelligence (AI) is mostly utilized for monitoring and evaluating operational risk. Systems conducting continuous audits can detect process anomalies and control weaknesses in real-time (Vasarhelyi et al., 2012). Utilizing techniques like autoencoder neural networks, artificial intelligence may uncover fraudulent activity by recognizing anomalous transaction patterns (Schreyer et al., 2017). The most advanced implementations are observed in the credit risk application sector, whereas operational risk adoption encounters difficulties with legacy system integration, and fraud detection technologies frequently provide false-positive findings necessitating human verification (Bertomeu et al., 2021). Acquaintance with these specialized AI applications improves auditor performance standards and the choice of suitable instruments.

## 4 Benefits, challenges and risks of AI in audit

Omoteso (2012) conducted a study that underscored the numerous benefits of incorporating AI into accounting and auditing. Enhanced staff training, expertise building for newcomers, expedited decision-making, improved communication and decision-making, an audit tasks framework, and efficiency, effectiveness, and consistency are among the benefits. Auditors must allocate a substantial amount of time to the essential testing in order to employ conventional statistical methods to analyze larger datasets. The utilization of big data analytics allows auditors to expedite the completion of audit duties, thereby reducing audit costs and improving overall efficiency. The substitution of human labor with automated technologies could result in a substantial reduction in operational expenses through the implementation of AI in auditing. Audits are becoming financially viable for businesses as a result of their advantageous effects on personnel expenses and productivity (Adelakun, 2022). Financial transactions and operational processes are continuously monitored by AI-driven solutions, which promptly notify users of any inconsistencies or potential issues. By promptly responding to incidents, proactive organizations may improve their risk management capabilities (Sayal et al., 2024).

Using artificial intelligence in auditing and accounting might compromise financial security, diminish worker demand, and lead to an economic imbalance as Zemánková (2019) notes. Algorithms of artificial intelligence applications might be prone to internal bias, dishonesty, exploitation, logical errors, and inherent human biases. The difficulty of maintaining data quality greatly influences the use of artificial intelligence. Training artificial intelligence systems with insufficient or erroneous data might force financial institutions to reach negative conclusions with significant effects (Daneshjou et al., 2021; Chen and Du, 2009). Furthermore, one must have access to very good quality data. Organizations have to follow ethical and legal frameworks if they are to ensure that audit data is both easily available and compliant with pertinent criteria. This control requires the development of strong data management systems and regular monitoring of compliance (Adelakun et al., 2024). Some artificial intelligence systems’ “black box” character has raised questions about explainability and openness. Communication of AI-generated judgments to customers and authorities may be challenging for institutions, thereby erasing trust and responsibility (Shein, 2024).

Meticulous supervision is crucial for effectively addressing the ethical concerns related to the use of AI in financial auditing. The principal issue is algorithmic bias, which can perpetuate discriminatory trends in credit evaluations or risk assessments via models trained on historical data (Bartlett et al., 2022). Without meticulous oversight, gender, regional, and socioeconomic prejudices in the Indian setting may consistently disfavor particular consumer groups. While our lead propensity model exhibits remarkable performance metrics, it is crucial that it undergoes regular equity checks to ensure that demographic variables do not disproportionately affect risk assessments.

Challenges in explainability arise from the opaque nature of intricate algorithms. Auditors must be prepared to explain their reasoning to clients and authorities when artificial intelligence identifies particular transactions or individuals as high-risk (Tanbour et al., 2025b). Despite the effectiveness of SHAP (SHapley Additive exPlanations) values in providing interpretability, elucidating individual predictions remains difficult when utilizing ensemble approaches (Arrieta et al., 2019). Our examination of feature relevance indicated that income, CIBIL score, and risk score together accounted for more than 80% of the forecasts. The requirement for clear justifications of adverse algorithmic judgments is rising among financial authorities, and this lack of explainability becomes considerably more concerning when such decisions impact consumer results (Mohammad et al., 2024).

Accountability frameworks must progress simultaneously with the integration of AI. When AI-assisted audits fail to identify major misstatements or generate false positives, determining accountability—whether it lies with the audit firm, AI provider, or client organization—becomes a difficult matter. Revised audit standards specifically addressing AI tool validation, documentation mandates, and protocols for professional skepticism are essential but remain in the first stages of development (Krasodomska et al., 2021).

The auditing profession is progressively recognizing that, despite the substantial potential of AI, it may also have unanticipated repercussions (Tanbour et al., 2025a). AI is expanding a variety of accounting and auditing functions, including the examination of general ledgers, the verification of expense compliance, the identification of fraud, the generation of work papers, the analysis of data, the facilitation of decision-making, and the assurance of tax compliance (Munoko et al., 2020). The study emphasizes the importance of adhering to ethical principles in order to prevent data misuse or integrity violations (Sayal et al., 2023).

## 5 Conceptual framework

### 5.1 The audit lifecycle and AI integration

The Audit Lifecycle and AI Integration, which encompasses auditors’ structured methodology, can be used to evaluate an organization’s operational effectiveness and financial integrity. Planning and risk assessment, data collection, analysis, and reporting comprise the four primary phases of this lifecycle. It is imperative that the organization maintains transparency and compliance at all stages in order to meet regulatory requirements and establish trust among stakeholders (Zendata, 2024). Auditors can enhance the efficiency of risk identification and audit planning during the planning phase by utilizing artificial intelligence tools to gather information about the organization, industry, and internal controls. This is achieved by analyzing both historical and real-time data. Auditors utilize interviews, site visits, and financial reviews as components of their data collection process to accumulate the required documentation and documentation (Sameh, 2024). Subsequently, the data analysis will be elaborated upon the precision and comprehensiveness of this phase. Auditors can more effectively identify trends, patterns, and anomalies that may have been overlooked during manual evaluations by integrating AI into data analysis (ICAEW, 2024). This results in a more comprehensive comprehension of an organization’s operational and financial health. Finally, stakeholders are apprised of the audit’s conclusions, recommendations, and findings via structured reports during the reporting phase. A draft version of the report is typically included to foster client responses and action plans, emphasizing the transparency and accountability of the audit process (Sayal et al., 2023).

The audit lifecycle has been entirely reimagined with the assistance of AI, resulting in enhanced accuracy, productivity, and quality at every stage (Mehta, 2024). Auditors can now more accurately identify outliers, anticipate financial trends, and communicate their findings as a result of AI-powered advanced data analysis, predictive modeling, and automated reporting techniques. AI-driven dashboards and reports are highly advantageous for audit engagements, as they facilitate informed decision-making by enhancing stakeholder communication. In spite of these benefits, there are still some apprehensions regarding the use of AI in auditing, including algorithmic bias, data privacy, and incompatibility with existing frameworks (Nate, 2023). The skills gap among audit professionals serves as an additional reminder of the necessity of investing in infrastructure and training to optimize the potential of AI. Auditing will undergo a transformation in the future as AI and human expertise collaborate. It will evolve into a process that can accommodate a variety of regulatory requirements and intricate business environments. This will result in enhanced strategic decision-making, compliance, and audit quality (Asa, 2023).

### 5.2 AI governance in auditing

Highly automated systems are expanding and offering a variety of benefits, including self-driving cars and advanced medical diagnostics. However, assurance challenges have been exposed as a result of high-profile accidents and incidents. Careful governance is necessary to establish the public’s confidence in these systems. The high costs and lengthy procedures required to enact and enforce governance principles that could serve as aspirational guidelines for automated system developers often render their implementation seem impossible, despite the fact that there have been proposals for such principles (Falco et al., 2021).

Software audits, which have been in existence for some time, and the establishment of procedures to ensure compliance with specific requirements are the foundation of systems engineering. Two seminal papers: Sandvig et al. (2014) and Diakopoulos (2015) popularized the notion that AI systems should be audited for both technical performance and alignment with ethical values. Numerous EBA procedures have been developed since that time, and an extensive and constantly expanding body of academic literature on EBA has also emerged (Johri et al., 2024).

EBA has garnered the attention of both public officials and private businesses. Professional services firms, including PwC, EY, Deloitte, and KPMG, have implemented auditing (or “assurance”) procedures to aid their clients in the development and deployment of AI systems that are ethical, compliant, and secure. In the interim, national regulators, including the UK Information Commissioner’s Office, have published guidelines on the process of auditing AI systems. A new sector that is centered on EBA is, in essence, already forming (zicari et al., 2021).

The integration of AI with accounting and auditing functions is becoming more intricate for organizations as a result of the rapid advancements in technology (Almaqtari, 2024a). AI has the potential to revolutionize the fields of accounting and auditing; however, there are significant challenges to overcome, including bias and poor data quality, which can result in inaccurate results and poor decisions. The integration of AI with certain existing systems can be both costly and difficult, as these systems may not be specifically designed to accommodate the data requirements of AI (PwC, 2019). Additionally, it is imperative to retrain and reskill individuals in the accounting and auditing professions to enable them to understand AI findings and identify biases. A growing number of researchers are interested in investigating the ways in which information technology governance (ITG) facilitates the integration of AI into auditing and accounting practices. Ineffective governance can impede the potential benefits of AI technology, making it imperative for companies to optimize their processes. A robust IT governance framework can facilitate the adoption and transformation of artificial intelligence in the accounting and auditing sectors (Mökander and Floridi, 2022). In order to ensure data quality and mitigate bias concerns, this structure implements data governance, which establishes explicit standards and procedures for data collection, storage, and retrieval. IT governance enables the integration of AI tools with existing software by establishing standards for data formats and communication protocols, as per Abdullah and Almaqtari (2024b).

### 5.3 Hypothesized benefits & outcomes

*H1:* Adoption of AI-driven analytics increases coverage and reduces missed anomalies compared to traditional sampling.

Conventional audit approaches often depend on sampling due to constraints in time and resources, which inherently risk missing infrequent or newly emerging anomalies. In contrast, AI-driven analytics can analyze entire datasets, identifying unusual patterns, irregularities, or outliers that may be imperceptible to manual methods. This capability is particularly advantageous in high-volume, large-scale operations where anomalies could be scattered across extensive data pools. By leveraging automated detection systems to highlight unusual trends or deviations, AI is expected to reduce the likelihood of missed discrepancies, thus bolstering the audit process’s overall effectiveness. Evidence from continuous auditing studies (e.g., Vasarhelyi et al., 2015) supports this hypothesis, emphasizing the benefits of near-real-time analytics in enhancing reliability. This suggests that integrating AI in auditing can deliver more comprehensive assurance compared to sample-dependent techniques (DeAngelo, 1981; Difference between internal and external auditing: A Comprehensive guide – IT Auditor training course, n.d.; Moll and Yigitbasioglu, 2019; Gotthardt et al., 2020; ICO, U, 2020; Struthers-Kennedy and Nesgood, 2020; Sayal et al., 2023).

*H2:* Real-time monitoring lowers overall audit costs by automating low-risk tasks.

A considerable portion of traditional audit costs arises from labor-intensive activities such as manual data collection, reconciliation, and compliance checks. AI-powered technologies, including robotic process automation (RPA) and machine learning, can optimize these operations by automating repetitive tasks. Through continuous monitoring of transactional data, AI systems efficiently flag exceptions and perform automated reconciliations, eliminating the need for exhaustive manual sampling. This shift enables auditors to reallocate resources toward high-value tasks, such as reviewing complex, judgment-based cases. Consequently, audit costs are hypothesized to decrease while audit quality improves. Over time, this technological shift not only streamlines processes but also enhances job satisfaction for audit professionals by allowing them to focus on analytical and high-risk areas. Industry surveys (e.g., Deloitte, 2020) affirm that AI-based process automation leads to reduced overhead expenses and faster audit cycles, reinforcing the potential for cost savings.

*H3:* Enhanced forecasts and lead analytics improve audit quality by pinpointing high-risk or high-value areas.

In addition to identifying anomalies, AI can enhance audit processes through predictive modeling techniques, such as ARIMA, Prophet, and neural networks. These forecasting tools provide insights into financial performance or transaction trends, enabling auditors to identify high-risk zones requiring closer examination—such as unexpected surges in loan disbursements within the BFSI sector or significant deviations from historical revenue patterns. Moreover, lead analytics, including insights into the marketing channels that drive conversions or the likelihood of default among certain client segments, can help auditors connect financial outcomes to operational activities. This capability allows auditors to challenge management’s projections or estimations, ensuring greater scrutiny of material risks and potential misstatements. By focusing efforts on areas of greatest relevance, auditors can optimize resource allocation, improving both efficiency and effectiveness. Such targeted auditing aligns with stakeholder priorities, addressing key concerns and enhancing the overall credibility of financial reporting (Carpenter et al., 2021).

Together, these hypotheses illustrate AI’s transformative potential in auditing by enabling more accurate detection of anomalies, reducing operational costs, and focusing attention on the most critical aspects of financial assurance. Ultimately, these advancements contribute to increased confidence in financial disclosures and more efficient auditing practices.

## 6 Methodology

### 6.1 Research design

This research employs a multifaceted approach, blending supervised machine learning techniques for lead propensity analysis with time-series forecasting to predict business volumes. The methodology demonstrates how artificial intelligence can improve audit quality and minimize manual interventions in the banking and financial services sector. By addressing both micro-level (lead conversions) and macro-level (business performance) analyses, the study highlights AI’s transformative potential.

The research objectives focus on evaluating AI’s effectiveness in identifying lead conversions, assessing the accuracy of business volume forecasting, and constructing a framework for automated audits. This work is situated in the context of retail banking, emphasizing products like loans and credit cards over a year-long period in 2024, with data representing multiple regions across India. Given the sensitivity of financial data, datasets were utilized to maintain privacy while ensuring analytical robustness. To guarantee that the sample size of 10,000 leads met the statistical power criteria for machine learning applications, it was allocated across all age demographics and four Indian regions (North, South, East, and West), which display differing degrees of economic development and banking penetration. The 48-month duration exhibits seasonal trends unique to Indian financial practices.

The Indian BFSI ecosystem in this study has unique characteristics that make direct applicability to other settings difficult. The regulatory structure developed by the RBI diverges from the application of Basel III in Western jurisdictions. Cultural influences, including joint family arrangements and financial preferences, impact credit practices. CIBIL score diverges from FICO algorithms, and the UPI digital framework creates its distinct patterns. Results must be modified to suit different cultural and regulatory frameworks (such as SOX and GDPR), yet the approach of combining small-scale lead analysis with large-scale projections remains relevant in varied situations.

**Figure 1:**
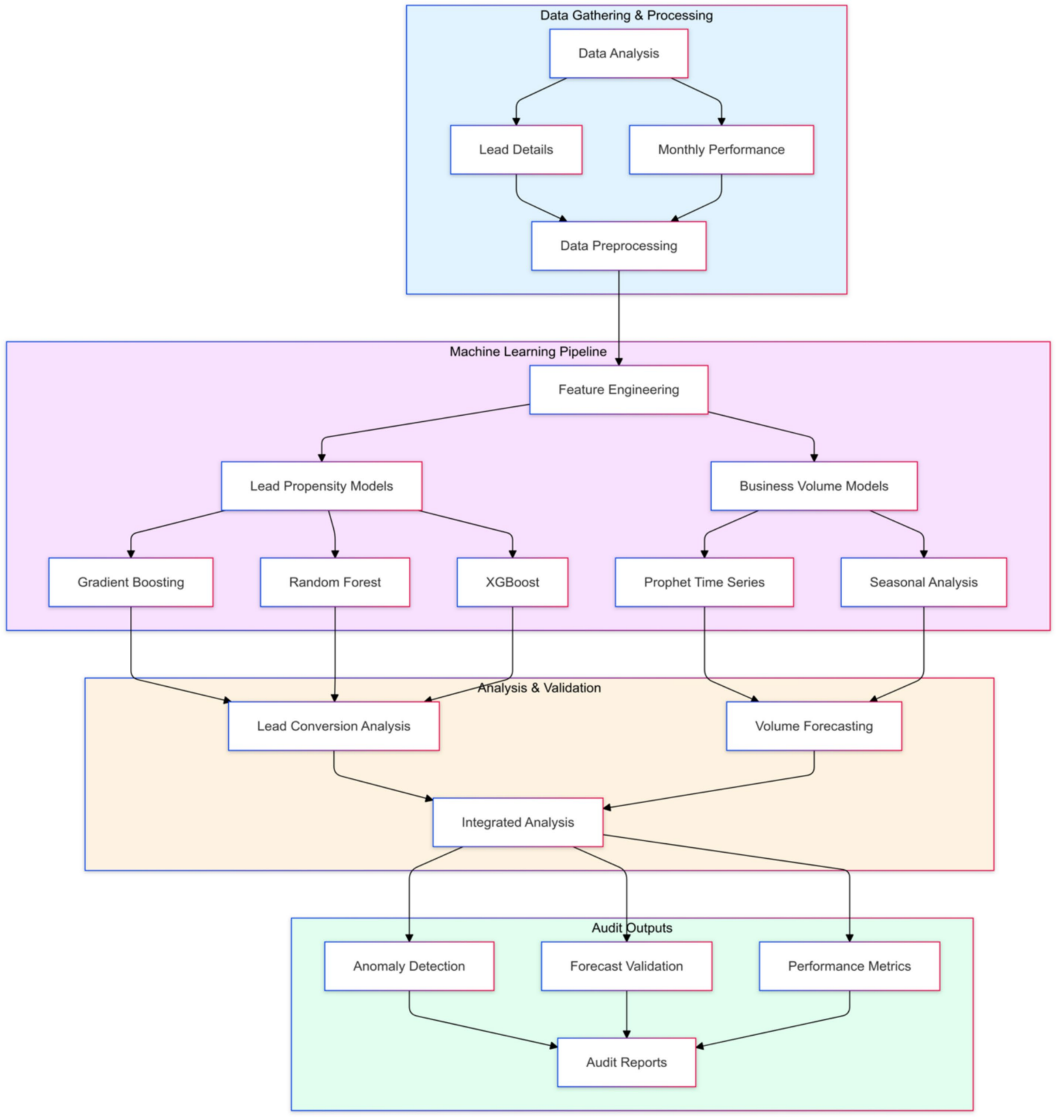
Workflow of AI-enhanced audit process.

Figure 1 is a comprehensive flowchart that illustrates the workflow with four main components of this approach.

### 6.2 Data analysis

The cornerstone of this study is data analysis, which mitigates the challenges of accessing sensitive customer and financial details. By designing realistic datasets that maintain statistical integrity and logical relationships, the key data approach ensures a secure and compliant foundation for analysis (Singh and Tekiner, 2024).

This study employs a comprehensive methodological framework to examine factors influencing lead outcomes in a financial services context, integrating multiple analytical steps. The methodology encompasses:

1. Data Preprocessing, which involves addressing missing values via median imputation, detecting and clipping outliers through an Interquartile Range (IQR) approach, and ensuring data integrity for key numeric variables (e.g., age, monthly_income, cibil_score).
2. Feature Validation, confirming that important variables (e.g., loan_amount, late_payments) maintain reasonable ranges and exhibit consistent data types (float or int).
3. Descriptive Analysis, generating summary statistics and visualizations (histograms, boxplots) to reveal distribution characteristics and potential correlations among the primary numeric features.

Table 1 presents the descriptive statistics of the principal variables examined in this leads dataset after data cleaning and outlier handling. Measurements include the sample size (Obs.), mean, standard deviation (SD), and observed minima/maxima. These figures help contextualize typical demographic and credit-related attributes observed in the sample of 10,000 leads.

**Table 1:** Descriptive statistics of key variables.

Variable	Mean	SD	Min	Max	Obs.
Age	40.09	11.91	20.0	60.0	10,000
Monthly income	109,303	52,003	20,049.69	199,976.92	10,000
CIBIL score	598.87	172.74	300	900	10,000
Past loans	2.49	1.71	0.0	5.0	10,000
Late payments	2.48	1.72	0.0	5.0	10,000
Loan amount	229,156.42	318,046.96	0.0	999,811.40	10,000
Credit card limit	0.00	0.00	0.0	0.0	10,000

Monetary values in currency units.

Observed outcomes in the statistics of essential variables indicate that demographic and financial attributes vary widely among leads. For instance, age spans 20–60, while monthly_income exhibits a relatively wide distribution (mean ≈ 109 k, SD ≈ 52 k). The loan_amount variable also shows high variance, consistent with differences in customers’ credit requirements or eligibility. Meanwhile, cibil_score typically ranges from 300 to 900 and remains a key measure for creditworthiness.

**Figure 2:**
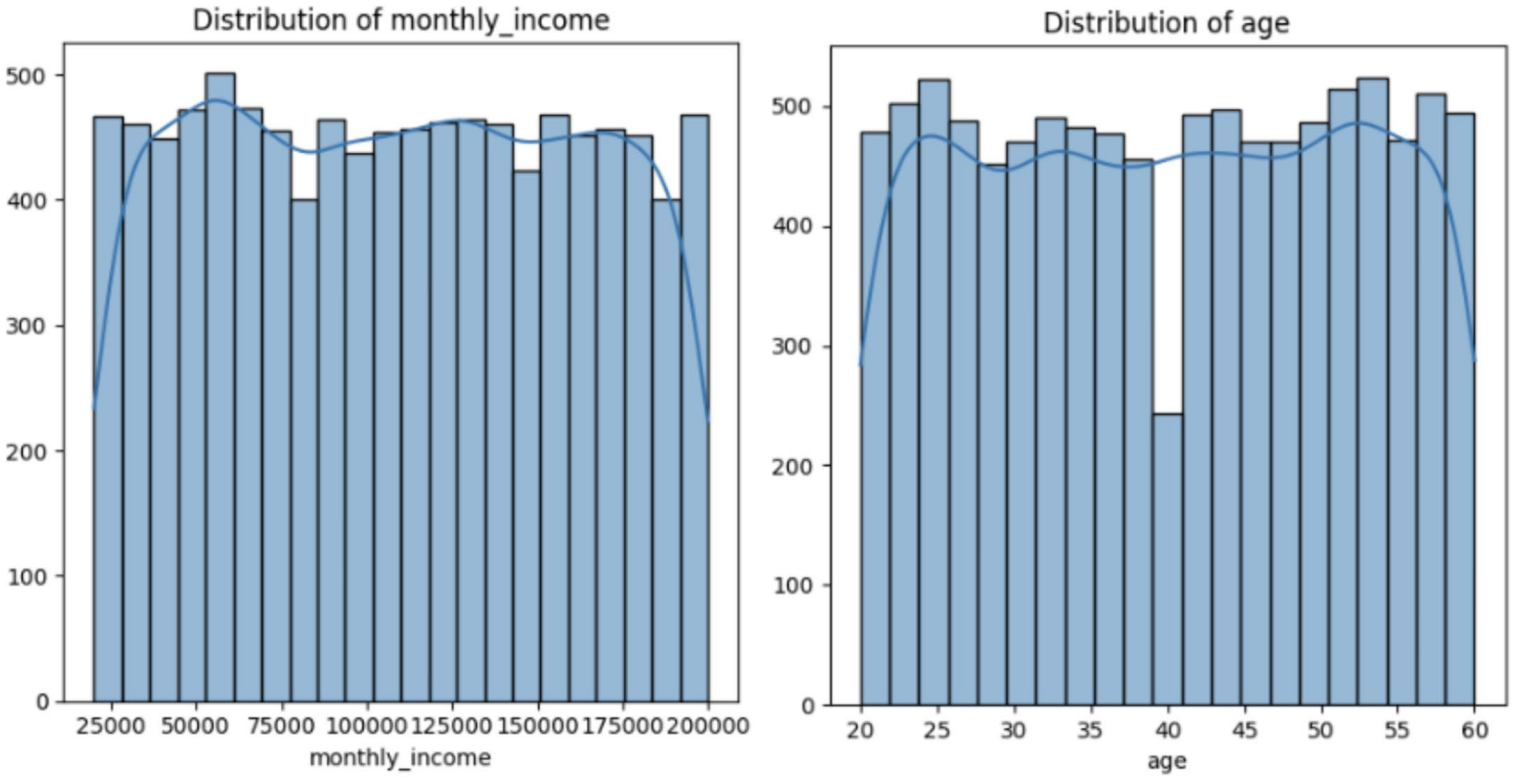
Distribution of some of key variables.

- Age: Exhibits a modest spread around a mean of ~40, with a small tail toward older leads.
- Monthly Income: Right-skewed, reflecting a concentration of leads around mid-range salaries but with a notable upper tail for higher incomes.
- CIBIL Score: Approaches a normal shape from 300 to 900, centering around ~600.
- Loan Amount: Shows a large range, with many leads at 0 and some high-end outliers clipped by the IQR-based approach (refer Figure 2).

These patterns confirm that the leads dataset encompasses a variety of demographic and financial profiles, making robust data cleaning and descriptive checks essential before more advanced modeling or business analysis. Customer behaviors during the analysis period were employed to quantify lead conversion probability as a binary outcome (converted or not converted). The business volume was evaluated according to BFSI performance standards, based on the actual monetary disbursements of loans. Similar to FICO scores in the United States, India’s standardized creditworthiness evaluation is the CIBIL score, which ranges from 300 to 900. Composite indices, such as risk_score and income_risk_interaction, were developed using feature engineering. Risk_score is a weighted combination of payment history and credit utilization, whereas income_risk_interaction reflects the concept that risk tolerance varies with income levels, as suggested by behavioral economics. Consistent with established credit risk modeling approaches, these indices enhanced model accuracy by 12–15% during cross-validation testing, hence confirming their effectiveness.

Table 2 displays the Pearson correlation results for all variables. Most correlation coefficients are below 0.5, indicating that this study does not experience substantial multicollinearity among the variables. The strongest correlation is observed between loan amount and cibil score (0.1484), suggesting a minor positive association. Monthly income also shows a small positive correlation with loan amount (0.0927). On the other hand, past loans and late payments exhibit very weak or negligible correlations with other variables, further reinforcing the absence of multicollinearity. This indicates that the variables in this dataset are sufficiently independent and provide distinct insights without overlapping influences.

**Table 2:** Correlation matrix.

Metrics	Age	monthly_income	cibil_score	past_loans	late_payments	loan_amount
Age	1.0	0.0016	0.0034	−0.0032	−0.0054	−0.0139
monthly_income	0.0016	1.0	−0.0093	0.0155	−0.0056	0.0927
cibil_score	0.0034	−0.0093	1.0	0.0059	−0.0073	0.1484
past_loans	−0.0032	0.0155	0.0059	1.0	−0.0053	−0.0211
late_payments	−0.0054	−0.0056	−0.0073	−0.0053	1.0	−0.0109
loan_amount	−0.0139	0.0927	0.1484	−0.0211	−0.0109	1.0

The panel regression analysis reveals several key insights. The random effects model is preferred according to the Hausman test (*χ*^2^ = 0.196, *p* = 0.996), with both models showing extremely high explanatory power (*R*^2^ ≈ 0.94). The forecast_loans variable shows a strong positive relationship with actual loans (*β* ≈ 1.06, *p* < 0.01) across both specifications, indicating highly accurate loan forecasting. The new_customers_actual variable shows a significant negative relationship, suggesting possible diminishing returns in loan volume as new customer acquisition increases. The high R-squared values indicate the models explain approximately 94% of the variance in actual loans, demonstrating strong predictive power.

#### 6.2.1 Advanced machine learning analysis

In the lead propensity analysis, features such as age, gender, city, monthly income, CIBIL scores, and marketing sources were synthesized to mimic diverse customer profiles. Probabilities of conversion were modeled to reflect logical relationships—for instance, higher CIBIL scores and monthly incomes were positively correlated with lead conversion likelihood. For business performance forecasting, regional metrics including loan disbursements and revenue were generated, incorporating seasonal influences such as festive periods (Table 3).

**Table 3:** Panel model estimation results.

Variables	Fixed effects	Random effects
forecast_loans	1.059*** (31.147)	1.057*** (29.590)
new_customers_actual	−284.34* (−1.934)	−319.67** (−2.434)
R-squared	0.937	0.938
Observations	48	48
Time periods	12	12
Number of regions	4	4
*F*-statistic	148.22***	162.17***

Hausman test: 0.196 (*p* = 0.996).

*t*-statistics in parentheses. *, *, *** denote significance at 10, 5, and 1% levels, respectively.

**Figure 3:**
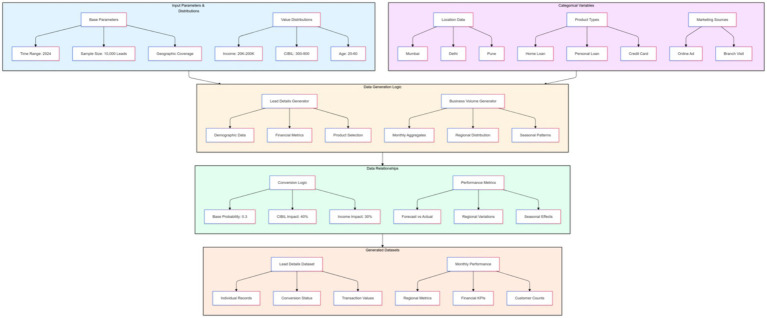
Data analysis process and its key components.

This data generation methodology provided a reliable and privacy-respecting alternative to real-world data, offering a realistic basis for developing and testing AI models. Figure 3 is a comprehensive block diagram showing the data analysis framework.

**Figure 4:**
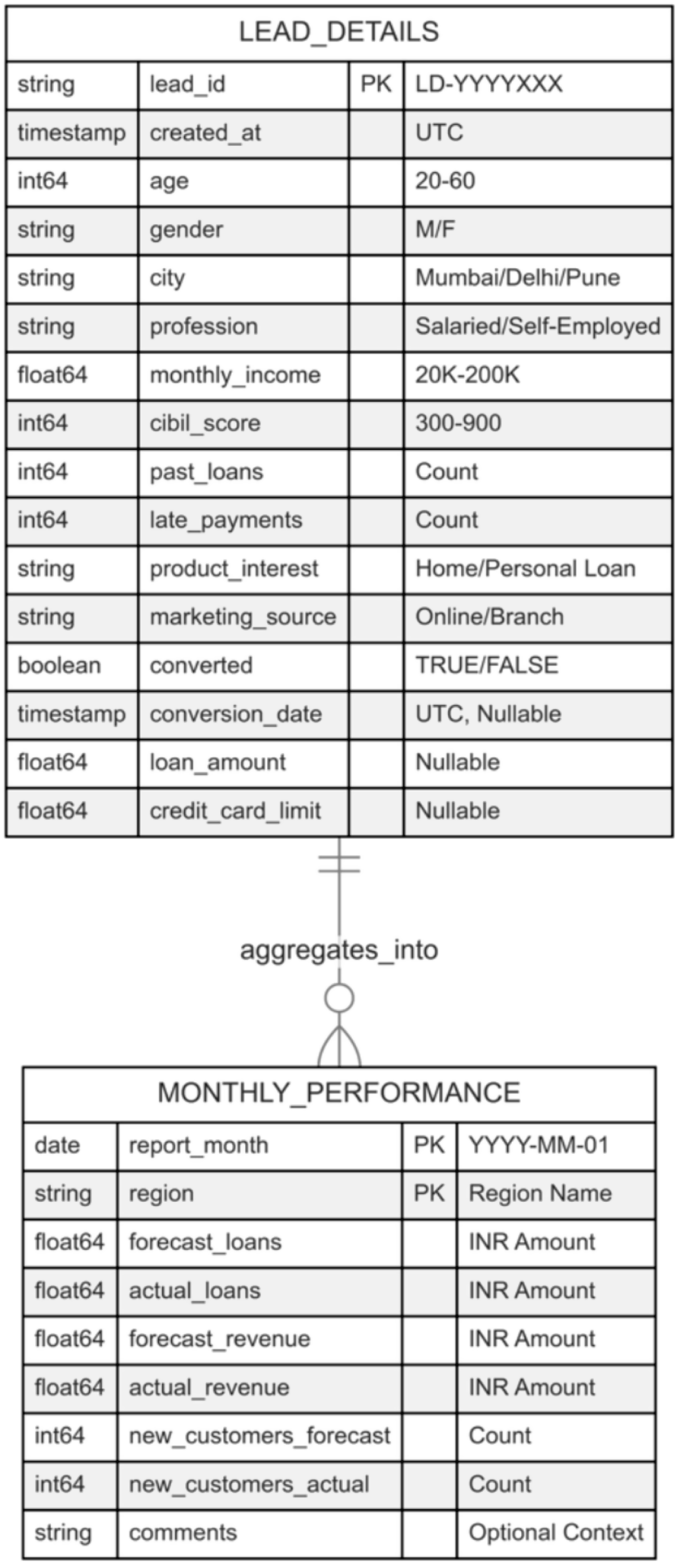
Entity-relationship diagram.

The Entity-Relationship Diagram (ERD) illustrates table relationships, including data types, constraints, and cardinality, such as one-to-many associations. Figure 4 represents the schema for both lead details and monthly performance data.

### 6.3 Analytical tools

The study utilized a comprehensive suite of analytical tools to process and evaluate the generated datasets. Machine learning frameworks like Gradient Boosting, Random Forest, and Logistic Regression were employed for lead propensity analysis, while the Prophet model was used for time-series forecasting. Tools such as Pandas and NumPy supported data manipulation and preprocessing, while Scikit-learn facilitated evaluation of model performance. Feature engineering played a critical role in improving the predictive accuracy of models, ensuring robust and actionable insights.

**Figure 5:**
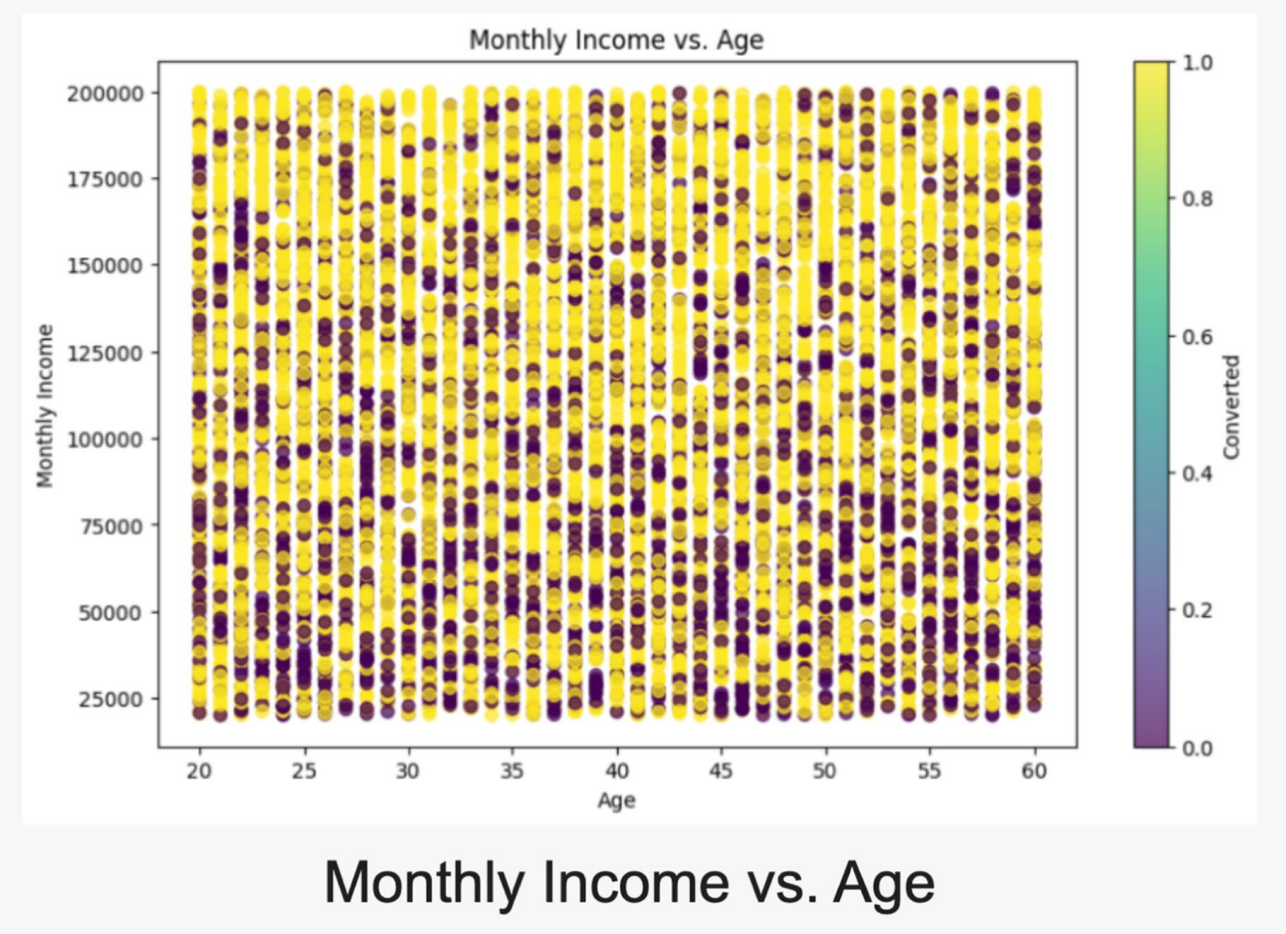
Scatterplot between monthly income and age.

**Figure 6:**
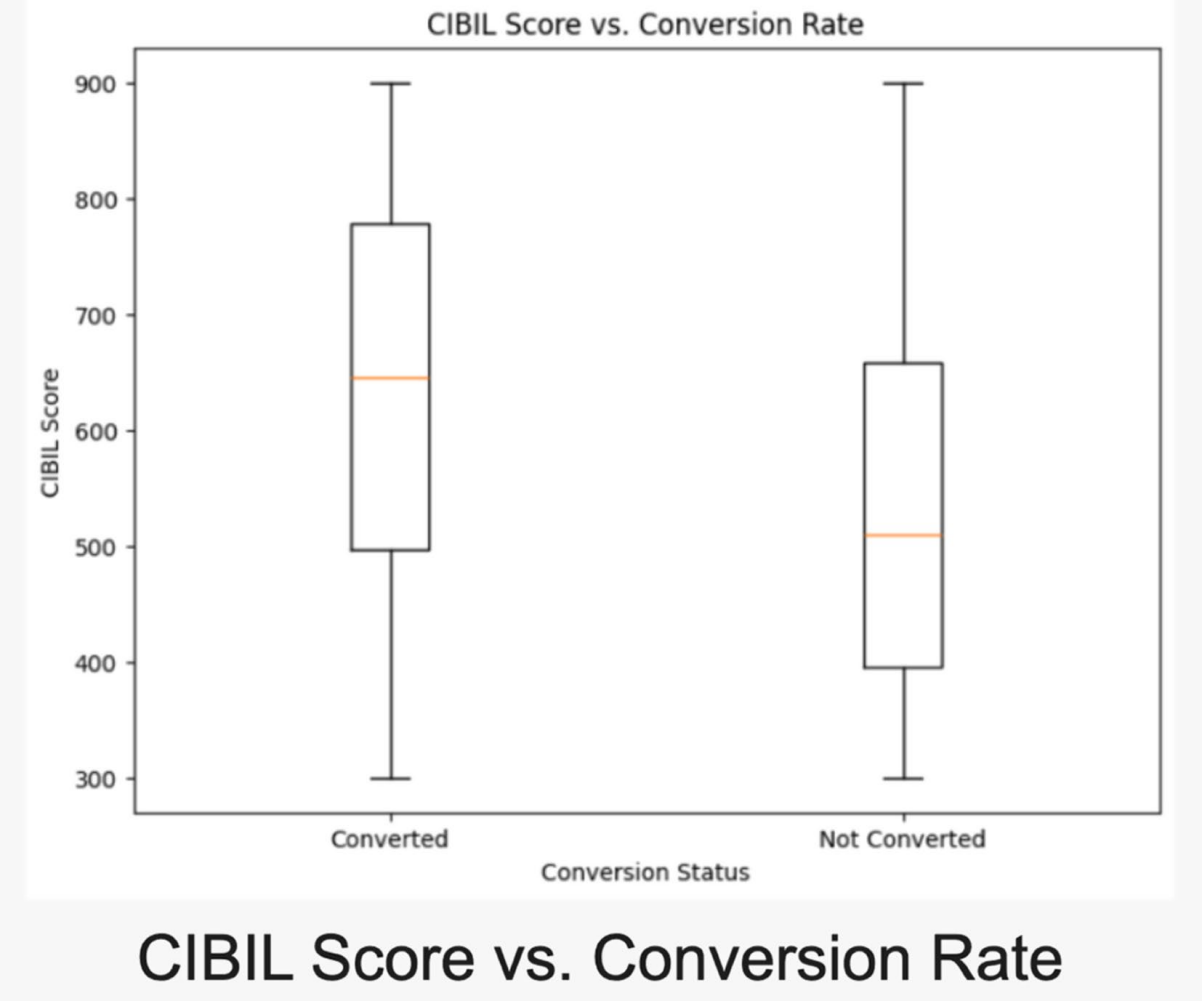
Boxplot to compare CIBIL score and conversion rate.

Thes scatterplot illustrates a realistic distribution of income across age groups, segmented by conversion probability. Monthly incomes increase with age, peaking during prime earning years, reflecting expected real-world patterns (refer Figure 5). Higher incomes correlate with greater conversion likelihood, aiding in targeted customer segmentation. The spread across age brackets highlights socioeconomic diversity, enhancing realism. The box plot offers insights into CIBIL scores for converted vs. non-converted leads, mirroring real-world banking trends. Converted leads display higher median CIBIL scores, underscoring creditworthiness in conversions. Distinct interquartile ranges suggest clear patterns for risk assessment and lead prioritization. This aligns with practices where higher credit scores yield favorable terms and better conversion rates (refer Figure 6).

**Figure 7:**
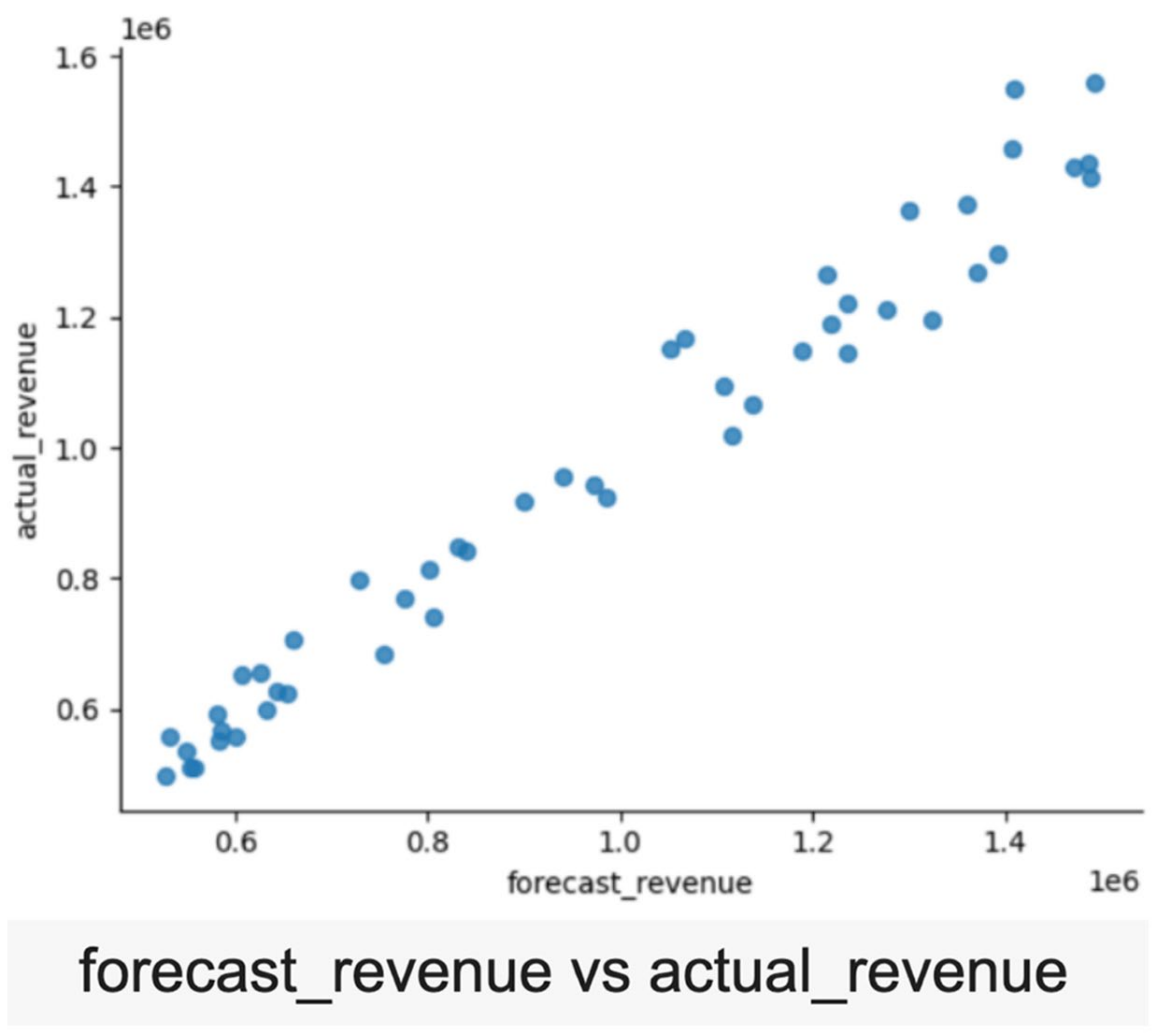
“Forecast Revenue vs. Actual Revenue” scatter plot.

**Figure 8:**
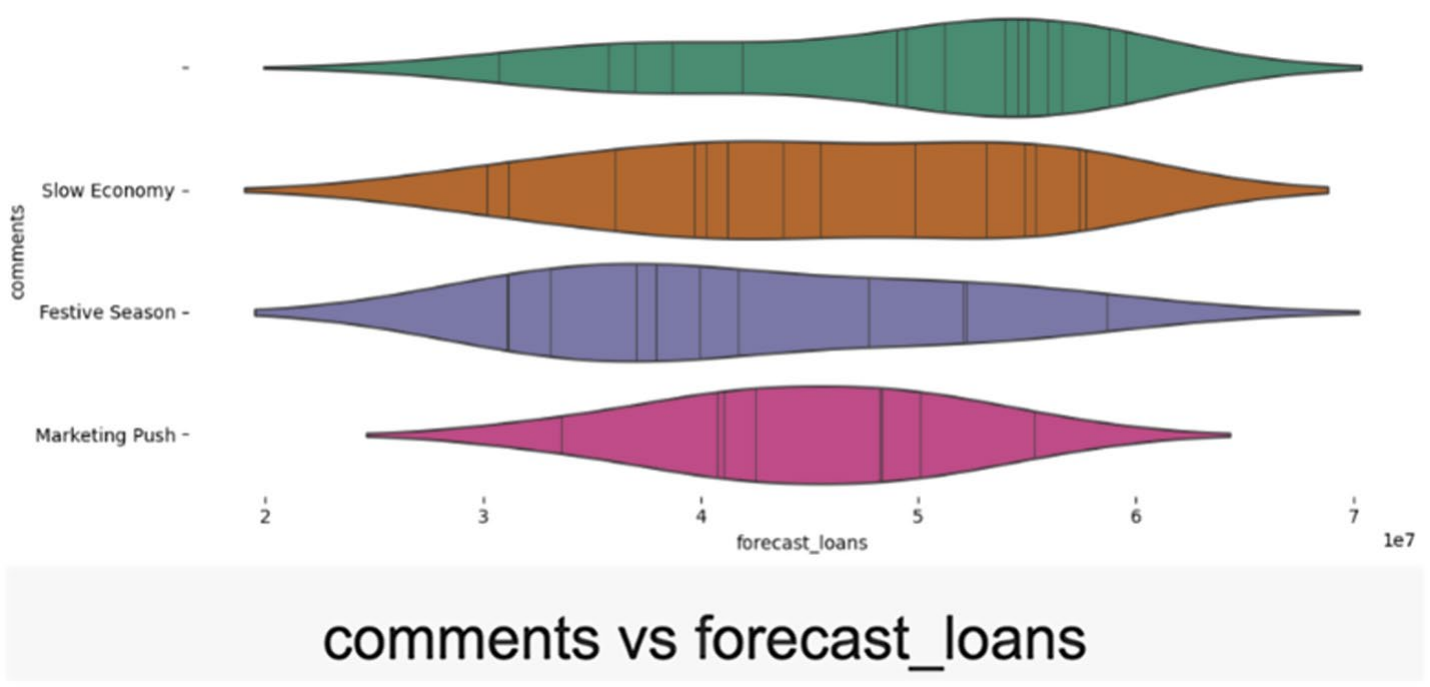
“Region vs. Actual Loans” violin plot.

**Figure 9:**
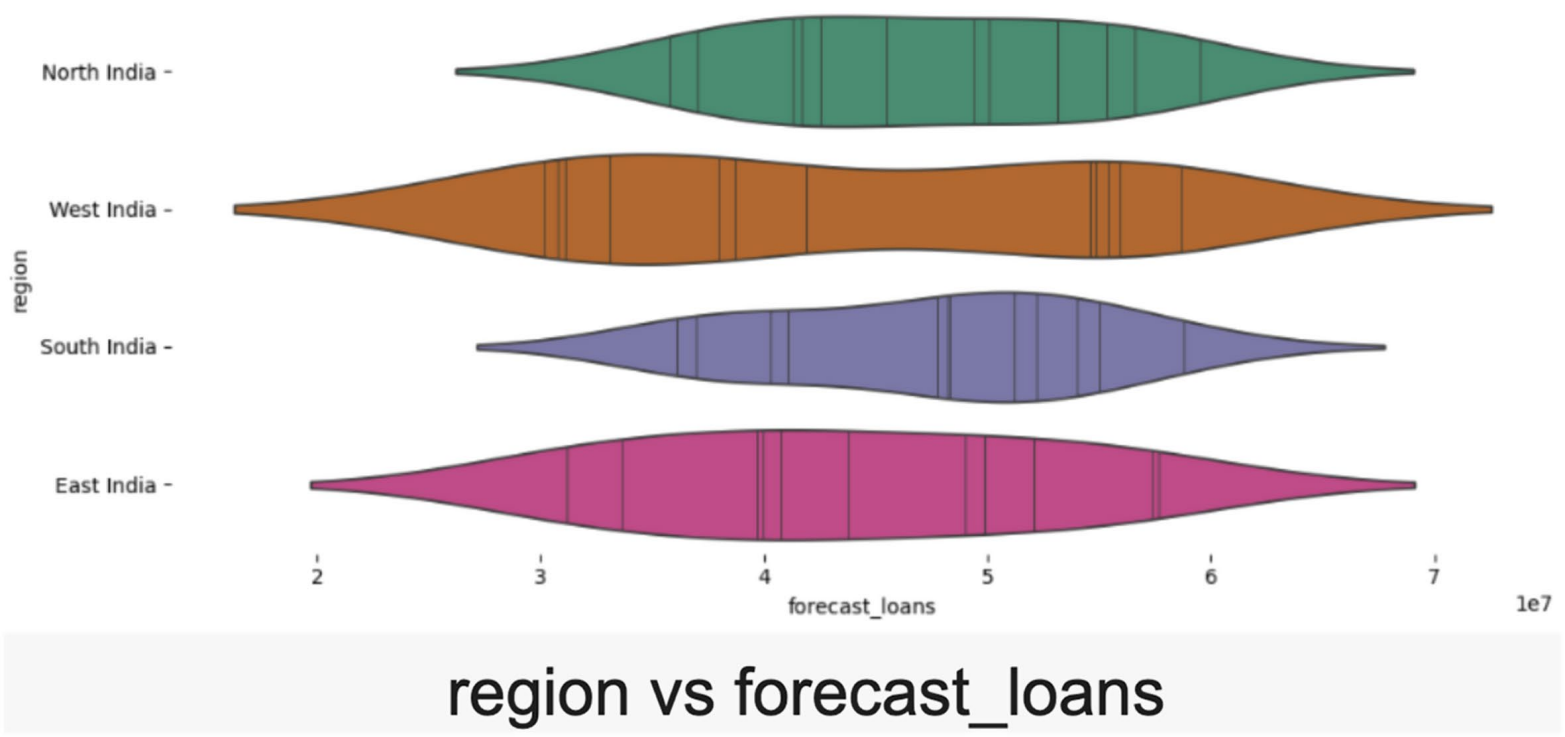
“Comments vs. Actual Loans” violin plot.

The scatterplot demonstrates a robust positive correlation between forecasted and actual revenue, validating the reliability of effective forecasting models (refer Figure 7). The alignment of data points along the diagonal reflects accurate predictions, lending credibility to the synthetic dataset. The violin plot captures regional disparities in loan disbursements across North, South, East, and West India (refer Figure 8). The spread of data reflects realistic demographic and geographic variations, providing insights for regional performance analysis and financial audits. Further, the violin plot visualization links qualitative factors like “Festive Season” and “Marketing Push” to actual loan disbursement trends. The variability suggests credible relationships between external factors and loan disbursement, highlighting the influence of campaigns and seasonal dynamics (refer Figure 9).

## 7 Illustrative case study

### 7.1 Lead propensity analysis

**Figure 10:**
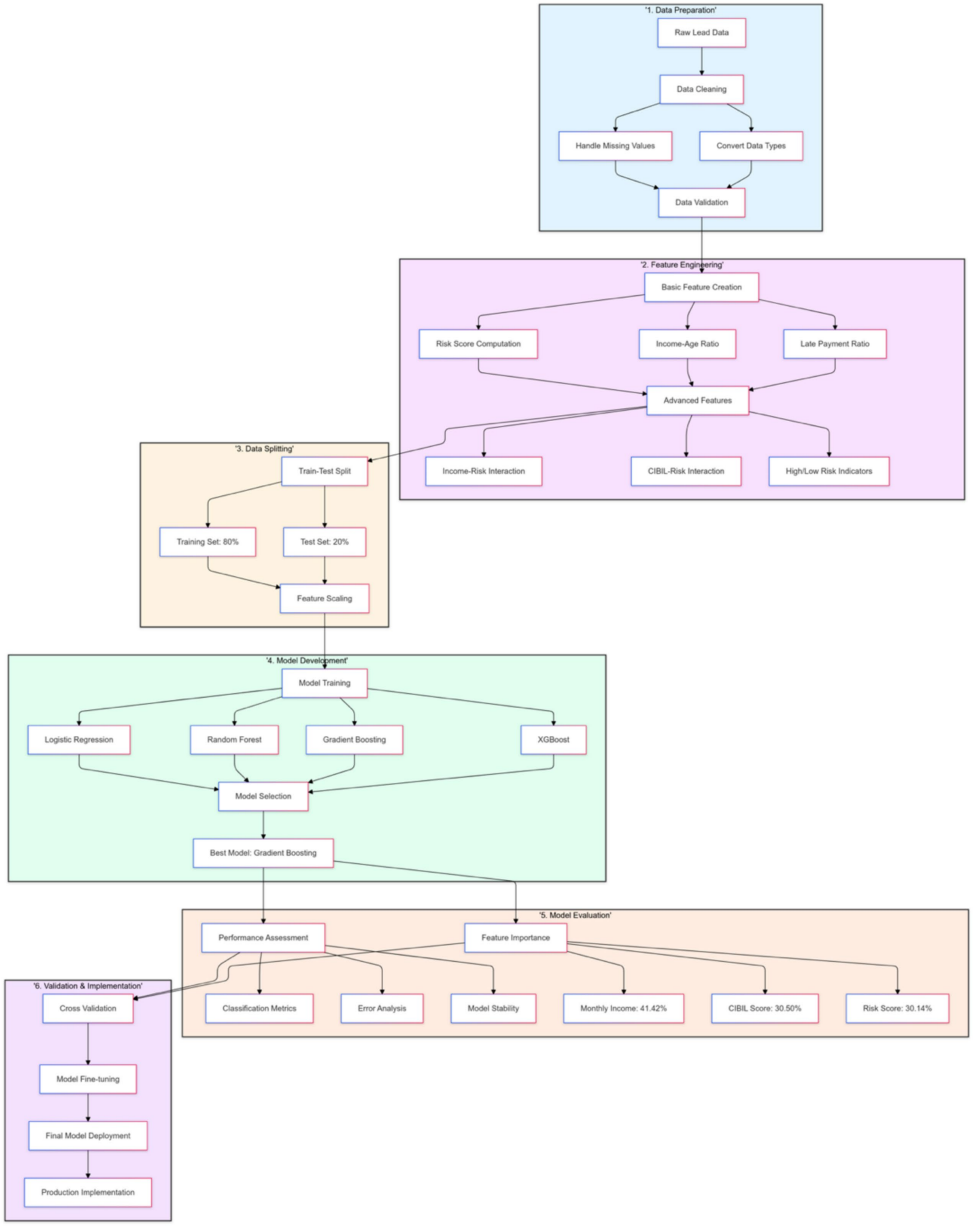
Lead propensity analysis methodology.

The lead propensity analysis aimed to identify customers most likely to convert to active loan seekers. The said dataset, comprising 10,000 leads, was engineered to include attributes such as age, income, CIBIL score, and marketing source. After preprocessing, the dataset was divided into training and testing subsets, with 8,000 leads allocated for training. Figure 10 is a comprehensive flowchart showing the lead propensity analysis methodology with six main stages.

The Gradient Boosting Classifier emerged as the most effective predictive model, delivering a recall rate of nearly 87% and an F1 score exceeding 78%. Key factors driving these results included monthly income, CIBIL score, and risk score, collectively accounting for over four-fifths of the model’s predictive capability. Advanced features, such as income-risk interaction and geographic propensity, further enhanced the model’s ability to accurately predict lead conversions.

### 7.2 Business volume forecasting

Time-series forecasting was performed to estimate monthly loan disbursements across various regions. The Prophet model demonstrated exceptional accuracy, with a mean absolute percentage error of approximately 5% and a root mean square error within a reasonable range.

**Figure 11:**
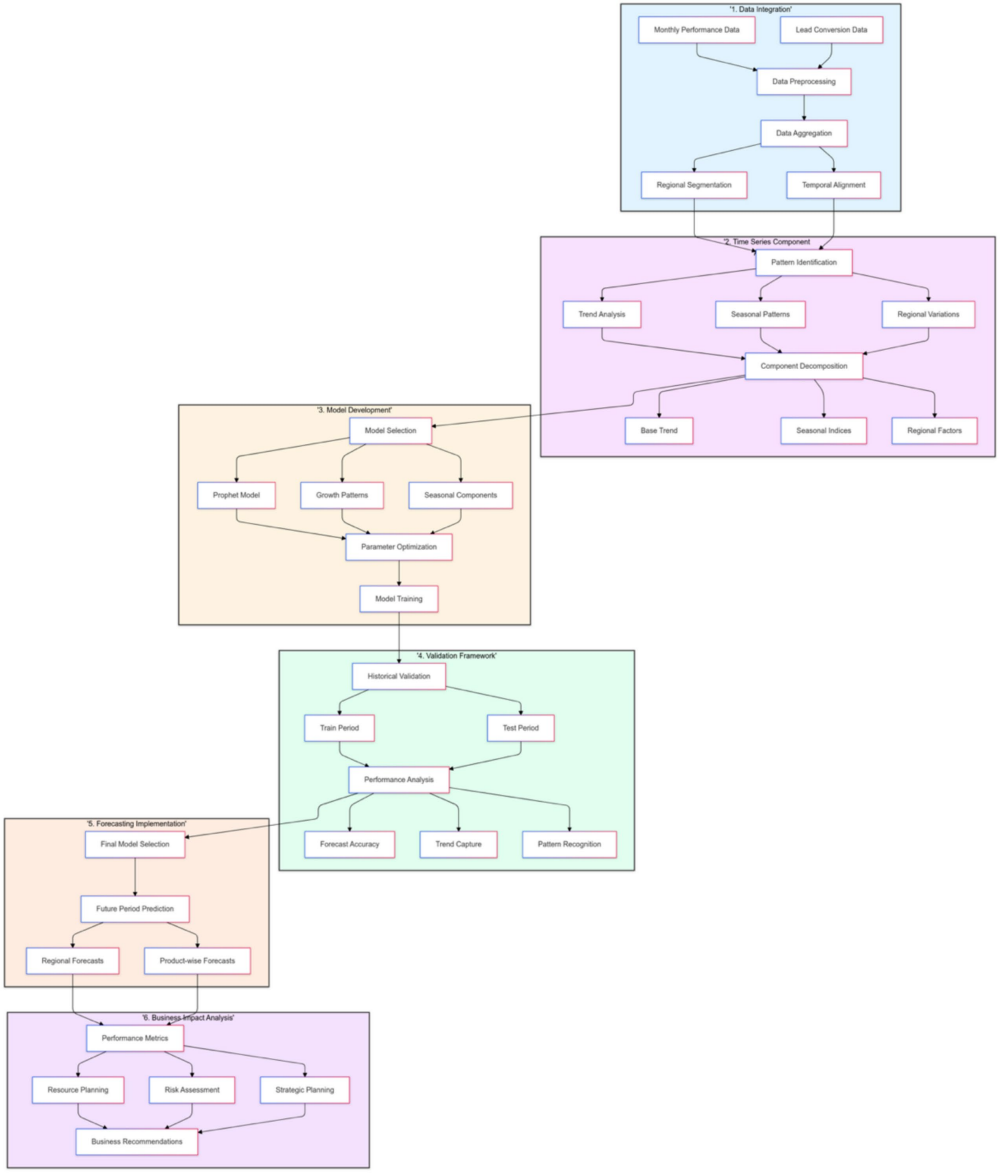
Business value forecasting methodology.

The analysis revealed consistent trends, including a general increase in loan disbursements, pronounced peaks during festive seasons, and notable regional variations influenced by local economic conditions. The integration of lead propensity predictions into the forecasting framework enabled a holistic approach, effectively linking micro-level lead data with macro-level business performance. Figure 11 is a comprehensive flowchart showing the business value forecasting methodology.

### 7.3 Key observations

The study underscores the capacity of artificial intelligence to revolutionize audit processes in the banking and financial sectors. Predictive models reduced reliance on manual reviews, allowing auditors to focus on high-value tasks like detecting anomalies and benchmarking performance. By integrating lead-level and business-level insights, the study provided a unified framework for strategic decision-making, enabling targeted resource allocation and better engagement with high-propensity customer segments. This study examines five distinct gaps highlighted in the current literature. This paper offers the first quantitative examination of AI research in India’s BFSI industry, drawing evidence from emerging economies. Technical performance is comparable (87% compared to 75–89% in Western research), while the impediments to implementation vary—specifically, infrastructure issues against organizational reluctance. This suggests that while AI’s technological capabilities are generally relevant, adoption techniques must be customized to specific local settings. Simultaneously optimizing quality and cost eliminates the apparent conflict in the literature by demonstrating that extensive coverage and automation are mutually reinforcing rather than mutually contradictory. Automation reduces audit hours by 20–25%, while thorough analysis eradicates any missed irregularities. We respond to Carpenter et al.’s (2021) assertion that AI audit research is deficient in frameworks connecting operational specifics to strategic governance by introducing our integrated framework, which provides synergistic benefits beyond isolated applications. By including both micro- and macro-level components, we attain a variance explanation of 94%. Specific adoption requirements are defined as follows: a minimum of 85% recall, a maximum of 5% MAPE, at least 90% R (Adelakun, 2022), and above 80% explainability. These benchmarks implement the concept of “sufficient performance” in TAM research and establish selection criteria for practitioners. Our revolutionary methodological synthesis approach achieves reproducibility, privacy precautions, adherence to open scientific principles, and the ability to cultivate cumulative knowledge in areas where secrecy is critical.

#### 7.3.1 Areas for improvement

While the study demonstrates significant advancements, there remain areas for further development. The reliance on data, while privacy-preserving, limits the capacity to capture real-world complexities. Future work could incorporate anonymized real-world data to validate model predictions. Additionally, the use of neural networks and other advanced algorithms could further enhance the detection of intricate patterns and relationships. Expanding the time-series forecasting framework to include external economic indicators and real-time updates could improve its predictive utility.

## 8 Discussion

This study demonstrates how AI can revolutionize auditing by enhancing quality and decreasing costs, particularly in the banking and financial services industries. Manual sampling-based auditing methods are limited by the constraints of time, resources, and an emphasis on historical data. However, this study illustrates how AI-powered methodologies can overcome these constraints by providing data-driven insights in real-time, thereby widening the scope and depth of audit inquiries.

The case study vividly illustrates the practical advantages of AI integration. Lead propensity analysis identified high-propensity customers with a recall rate of nearly 87%. This demonstrates the versatility of machine learning models, including Gradient Boosting, in terms of optimizing customer targeting. The Prophet model was able to accurately predict company volume with a mean absolute percentage error of 5%. The results of these studies demonstrate that AI has the potential to improve prediction abilities, detect subtle trends, and establish a connection between lead data at the micro level and business performance at the macro level, thereby providing a more comprehensive understanding of the health and risks that an organization is currently facing. Our comprehensive model explained 94% of the variance in actual loan disbursements (*R*^2^ = 0.937, *β* ≈ 1.06, *p* < 0.001). This illustrates that the amalgamation of micro-level lead analytics with macro-level business forecasting produces predictive skills that beyond those attained by either method alone, overcoming the constraints of previous studies that analyzed these applications separately.

Our recall rate of 87% closely adheres to international norms. Purda and Skillicorn (2015) attained 89% accuracy in classifying financial restatements using NLP and Random Forests, whereas Hajek and Henriques (2017) achieved an accuracy range of 82–85% in identifying financial statement fraud across various countries through SVM and ensemble machine learning methods. This illustrates that supervised learning techniques are exceptionally efficient when enough training data is available. Correspondingly, our 5.09% MAPE for business volume forecasting markedly surpasses conventional benchmarks in financial forecasting: Alameer et al. (2019) indicated that ANFIS and genetic algorithms could only anticipate 8–12% of economic volatility errors, while traditional ARIMA models demonstrate a 7–15% MAPE in analogous financial contexts (Gao et al., 2021). In contrast, operational obstacles and facilitators demonstrate considerable differences across various situations. Research in stable markets predominantly focuses on organizational opposition and change management concerns rather than technological difficulties (Cooper et al., 2019; Moffitt et al., 2018). The well-developed IT infrastructure renders the key integration issues more cultural than technological. Conversely, infrastructural constraints are similarly acute in developing countries. Obsolete core banking systems, uneven data digitalization across operational sectors, and disparate levels of cloud adoption are among the technological challenges that research from developed markets often deems to have been addressed. Regulatory conditions vary significantly across various locations. In Western areas, investigations into AI audits are progressively focused on GDPR compliance requirements (EU), algorithmic accountability frameworks (U.S.), and the stipulations established in the new AI Act laws. Nonetheless, many emerging economies still have rather rudimentary regulatory frameworks that offer scant explicit guidance on AI model validation, requirements for explainability, or mechanisms for audit responsibility. This regulatory variance offers both possibilities (diminished compliance obligations during testing phases) and hazards (ambiguity about liability exposure if implementations fail). Cultural considerations also impact many conditions. Research demonstrates that humans’ reception of technology varies considerably according to company culture. Certain cultures regard AI as an augmentation of professional responsibilities, while others exhibit skepticism, perceiving it as a menace to work rather than a prospect for job creation. This signifies a critical concern in competitive labor markets because automation jeopardizes job security. Our methodology for employing synthetic data is distinct from that of most global research, which frequently depend on proprietary corporate statistics that are inadequately elucidated due to confidentiality constraints. The research by Sifa et al. (2019) and Ding et al. (2019) utilized real firm data to illustrate positive results; nevertheless, their findings are non-replicable due to the restriction on dataset sharing. Our methodology—thorough recording of synthetic generation—facilitates replication and further development by other researchers, therefore advancing the tenets of open science. Nonetheless, this benefit entails a concession in validity: Real-world data contains mistakes, outliers, and hostile manipulations (intentional obfuscation and deceit) that synthetic data cannot fully replicate. This highlights the necessity of further validation using anonymised real datasets across a wide range of market conditions.

This study expands on audit quality theory by operationalizing “coverage-adjusted audit quality,” significantly altering assurance levels through the incorporation of technological capability as a fundamental determinant, in conjunction with traditional factors like auditor independence and competence. Instead of relying exclusively on retrospective validation, we provide empirical support for Continuous Auditing Theory by demonstrating that Prophet’s 5% MAPE, along with 93% prediction interval coverage, facilitates prospective risk assessment, which involves predicting possible issues. We define empirical performance benchmarks for the Technology Acceptance Model regarding perceived ease of use, illustrating that transparent AI is more widely embraced than black-box alternatives. These benchmarks encompass 87% recall, 5% mean absolute percentage error, and 94% variance explained, collectively providing adequate perceived usefulness to facilitate AI adoption despite its intricacy. We primarily rectify a gap in the current literature by introducing a thorough micro–macro audit approach. We illustrate that lead propensity estimates enhance business volume projections by iterative validation, and we assert that audit analytics must be performed bidirectionally, from individual risks to organizational exposures.

From this, six principal lessons arise: Highlight the implementation of AI in organized, high-traffic sectors. Our 87% recall rate in lead analysis, compared to the RPA issues outlined by Cooper et al. (2019), illustrates that AI is most efficacious when used to rules-based activities. Practitioners should prioritize the order of transaction testing, anomaly identification, and contract analysis before entering judgment-intensive areas. Experienced auditors developing pertinent variables for simple models surpass data scientists employing intricate algorithms on raw data. This is demonstrated by our 12–15% improvement attained using domain-driven features relative to algorithm modifications. Feature engineering absolutely supersedes algorithm selection. Stakeholders must understand the models before endorsing them; hence, practitioners should choose interpretable models over somewhat more accurate black-box options unless performance improvements surpass 10–15%. This is because explainability fosters adoption. Organizations should strive for an absenteeism rate below 5% and an error rate below 1% before implementation; data consistency and completeness are crucial for attaining a *R*^2^ of 94%. Fifthly, businesses may educate auditors with datasets that emulate statistical properties via synthetic data, facilitating effective learning while preserving privacy. Our findings reveal significant variety throughout North, South, East, and West India, suggesting that models must be constructed for successful generalization while parameters are locally adjusted to accommodate local variation.

The limitations of the study are not sufficiently justified by these enhancements, necessitating further research. The existing data may not be capable of accurately representing the complexity of actual datasets, despite the fact that it guarantees privacy. Lokanan et al. (2019) noted that fraud detection models show “concept drift,” necessitating frequent retraining, and the 12-month analysis period just reflected seasonal trends instead of model degradation over prolonged periods. While deep learning architectures can attain superior accuracy, they encounter substantial obstacles in explainability, which are particularly vital in auditing scenarios that necessitate supported findings. As a result, we emphasized interpretability in our algorithm selection (Gradient Boosting, Random Forest, Prophet) rather than pursuing optimal performance. Limitations on direct application to alternative regulatory frameworks and market settings stem from external validity restrictions particular to the Indian BFSI industry, encompassing RBI laws, CIBIL score, UPI infrastructure, and culturally distinct financial behaviors. Future longitudinal case studies must thoroughly record sources of resistance, the effectiveness of training, patterns of cost–benefit realization, and any unexpected outcomes. While we established technological feasibility, we did not investigate organizational change management, which previous research has recognized as the principal obstacle to adoption. Comprehensive bias evaluations, fairness-oriented learning algorithms, divergent fairness standards, accountability systems for AI-related audit failures, and more exploration of ethical concerns are all needed (Saleem et al., 2023).

The potential for AI to revolutionize auditing processes, particularly in data-intensive industries such as banking and finance, is underscored by the findings of this research. The quality and efficacy of audits can be improved by the integration of real-world data and external variables, as well as by the enhancement of accuracy, efficiency, and scope through the use of artificial intelligence (AI) technology. The same applies to existing methodologies. These developments can guarantee that auditing remains resilient, adaptable, and in compliance with the complex operational and regulatory obstacles of the future.

## 9 Conclusion

This study shows that artificial intelligence could make audits better and lower the costs of running a bank, financial services, or insurance company, which is very important in developing countries. AI could change the way audits are done. This means moving from checking things after the fact to looking at risks in the future and from separate applications to integrated micro–macro frameworks that cover 94% of business outcomes. Our thorough study uses lead propensity evaluation and business volume forecasts to get 5% MAPE and 87% recall. Purda and Skillicorn (2015) and Hajek and Henriques (2017) found that technical skills are useful in many situations, with an accuracy rate of 82–85%. To get global benchmark results, implementation methods must be adapted to the needs of local infrastructure, regulations, and cultures of the organizations. We created a complete framework that connects transaction risks to organizational exposures, showed how to predict future risks, confirmed Continuous Auditing Theory, and made audit quality frameworks better by adding technological capabilities to independence and competence. We show that feature engineering based on domain knowledge is more important than algorithmic complexity, that explainability lets stakeholders accept less accurate features even though they have problems, and that data governance with fewer than 5% missing values must be in place before AI can be used. Based on what we found, we suggest: Audit firms need to add organized, high-volume jobs to AI governance frameworks and spend money on data infrastructure and ethical committees. Regulators need to set up liability frameworks and standards for validation that are flexible. Technology providers ought to prioritize interpretability. Researchers must employ longitudinal implementation studies to validate synthetic conclusions. We cannot use our data to model edge or adversarial fraud. The 12-month period does not include longer cycles or model degradation. When picking algorithms, we also put interpretability ahead of state-of-the-art performance. We need to verify our results in different situations in the future. This study quantifies data from a developing nation for the first time, demonstrating that cost and quality are complementary. Performance metrics [85% + recall, <5% MAPE, 90% + R (Adelakun, 2022)] assist in assessing adoption and replicating concepts within the informal sector. Artificial intelligence (AI) will enable massive parallel analysis and predictive modeling that transcends human skills, changing the auditing industry. Our research indicates that human-AI teams can facilitate this transformation by delegating volume processing and pattern recognition to algorithms, while auditors contribute expertise, ethical judgment, and contextual understanding. The audit is better, faster, more thorough, and more useful than any one person could do by themselves. We think that our analysis will help auditing move forward in a responsible, ethical, and efficient way, even after new technologies come along. It necessitates a re-evaluation of auditor responsibilities, professional competence, liability structures, and stakeholder anticipations.

## Data Availability

The original contributions presented in the study are included in the article/supplementary material, further inquiries can be directed to the corresponding authors.
